# Eutrophication and Warming Drive Algal Community Shifts in Synchronised Time Series of Experimental Lakes

**DOI:** 10.1111/1462-2920.70159

**Published:** 2025-07-24

**Authors:** Rebecca E. Garner, Zofia E. Taranu, Scott N. Higgins, Michael J. Paterson, Irene Gregory‐Eaves, David A. Walsh

**Affiliations:** ^1^ Department of Biology Concordia University Montreal Quebec Canada; ^2^ Innovative Genomics Institute University of California, Berkeley Berkeley California USA; ^3^ Groupe de recherche interuniversitaire en limnologie Quebec Canada; ^4^ Environment and Climate Change Canada Montreal Quebec Canada; ^5^ IISD Experimental Lakes Area Winnipeg Manitoba Canada; ^6^ Department of Biology McGill University Montreal Quebec Canada

## Abstract

Lake ecosystems are increasingly impacted by eutrophication and climate change. Whole‐lake experiments have provided ecosystem‐scale insights into the effects of freshwater stressors, yet these are constrained to the duration of monitoring programmes. Here, we leveraged multidecadal monitoring records and century‐scale paleogenetic reconstructions for experimentally fertilised and unmanipulated lakes in the IISD Experimental Lakes Area of northwestern Ontario, Canada, to evaluate the responses of algal communities to nutrient and air temperature variation. We first validated the paleogenetic analysis of sediment DNA by demonstrating the synchrony of algal community changes with monitoring records. Algal communities underwent significant compositional shifts across experimental nutrient loading regimes and climate periods, with baseline assemblages informed by paleogenetics. Nonlinear regression modelling of algal community change in monitoring and paleogenetic time series showed the expected response that nutrients were strong drivers in fertilised lakes. Paleogenetic records reflected the century‐scale impacts of climate warming and its combined effects with eutrophication, previously underestimated by monitoring. The synergy between eutrophication and warming points to eutrophic priming of the food web to respond to rising temperatures. Overall, the paleogenetic integration of algal diversity across habitats and seasons enables the detection of slow‐acting climate change on lake ecosystems increasingly altered by nutrient pollution.

## Graphical abstract

Algal community dynamics recorded by traditional monitoring and paleogenetic analysis of sediment DNA archives of experimentally fertilised and unmanipulated lakes in the IISD Experimental Lakes Area exposed synergistic effects of eutrophication and climate warming.

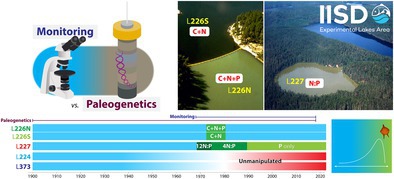

## 1 Introduction

Eutrophication is a pervasive water quality issue intensified by climate change (Jeppesen et al. 2014; Rodgers 2021). Algal blooms fuelled by excess nutrients create anoxic dead zones, displace fish and invertebrate habitats, and disrupt ecosystem services such as safe drinking water and recreation. As fertiliser applications increase to meet global demands for food and biofuels, eutrophication is predicted to become more severe and widespread (Sinha et al. 2017). In many regions, climate change may exacerbate terrestrial runoff through increased precipitation, affecting how lakes function as sinks or sources of nitrogen (N), phosphorus (Wu et al. 2022) (P), and carbon (Tranvik et al. 2009) (C). In altering the strength and frequency of water column stratification, climate warming can indirectly prolong and intensify eutrophication via internal nutrient loading, even when terrestrial runoff is reduced (Søndergaard et al. 2003). Eutrophication in turn feeds back into climate change, when greenhouse gases produced during algal bloom senescence are released to the atmosphere (Hessen et al. 2024).

In its more than 50 years of operation, the Experimental Lakes Area (ELA; now the International Institute for Sustainable Development [IISD] ELA), located in the Boreal Shield ecozone of central Canada, has contributed foundational knowledge on freshwater stressors through the experimental manipulation and monitoring of whole lake ecosystems. Initiated in 1969, the ongoing experimental nutrient loading of Lake (L)227 has sustained elevated algal biomass at decreasing N:P ratios while maintaining consistent P loads, even after N addition ceased in 1990 (Higgins et al. 2018). The initial results of the L227 experiment demonstrated that carbon limitation did not control annual algal production and later demonstrated the ineffectiveness of N remediation in controlling the magnitude of algal blooms (Higgins et al. 2018; Paterson et al. 2011; Schindler et al. 2016). Another whole‐lake experiment performed in the two basins of L226 also provided compelling evidence to policymakers that eutrophication management should focus on P abatement: the northeast basin (L226N) received experimental C, N, and P additions and experienced several‐fold greater algal growth relative to the southeast basin (L226S) that received only C and N (Schindler et al. 2016; Findlay and Kasian 1987). L226 recovered to oligotrophic status after 7 years of nutrient loads ceased in 1980 (Turner et al. 2005). Since 1969, the ELA has also monitored meteorological conditions and limnological data from several unmanipulated reference lakes, which have been useful for tracking the effects of climate change (Findlay et al. 2001).

Long‐term lake monitoring programs are generally rare, although there are a growing number of lakes around the world with several decades of regular data collection (Hampton et al. 2008; Yang et al. 2016; Jochimsen et al. 2013; Goldman et al. 1989; Arhonditsis et al. 2004; Bailey‐Watts et al. 1990; Lathrop et al. 1996; Fukushima and Arai 2015). Often, monitoring is initiated after ecosystems are observably impacted, concealing pre‐impact conditions from the lake record (Smol 2019). The ELA is exceptional in its long‐term monitoring of whole‐lake experiments and unmanipulated reference lakes (Stokstad 2008). Yet, more substantial pre‐manipulation records are needed to anchor the trajectories of the ELA systems to baseline conditions. Paleolimnological techniques were previously used to reconstruct algal assemblages in the fertilised and unmanipulated lakes based on classical proxies limited to select groups with subfossils (Zeeb et al. 1994; Enache et al. 2011) or pigments (Leavitt and Findlay 1994; Cottingham et al. 2000) preserved in sediments. In a recent advance, the paleogenetic analysis of DNA archived in buried cells or bound to sediment particles traces species without morphological remains (Domaizon et al. 2017; Capo et al. 2021). Applying a paleogenetic approach thus enables the reconstruction of a broader lake food web on scales of observation relevant for detecting slow‐acting ecosystem stressors (Smol 2019; Davidson and Jeppesen 2013). Progress has been made in this area by studying the multiple‐stressor responses of microeukaryotes in the paleogenetic record (Capo et al. 2017; Keck et al. 2020; Ibrahim et al. 2021; Barouillet et al. 2023).

In this study, we paired multidecadal monitoring and ~120‐year paleogenetic records of experimentally fertilised and unmanipulated lakes in the ELA to identify drivers of eukaryotic algal community composition. We first demonstrated congruence between the temporal dynamics of algal assemblages recorded in traditional taxonomic monitoring with sediment DNA time series. Leveraging the different timescales and resolutions presented by monitoring and paleogenetics, we modelled the contributions of eutrophication and warming to algal community changes in experimentally fertilised lakes and unmanipulated lakes that were primarily influenced by climate. Overall, our observations show that sediment archives provide a more comprehensive long‐term perspective on lake ecosystems, capturing signals from both fast‐ and slow‐acting stressors and their synergies, which are often obscured from traditional monitoring programmes.

## 2 Experimental Procedures

### 2.1 Lake Experiments

**FIGURE 1:**
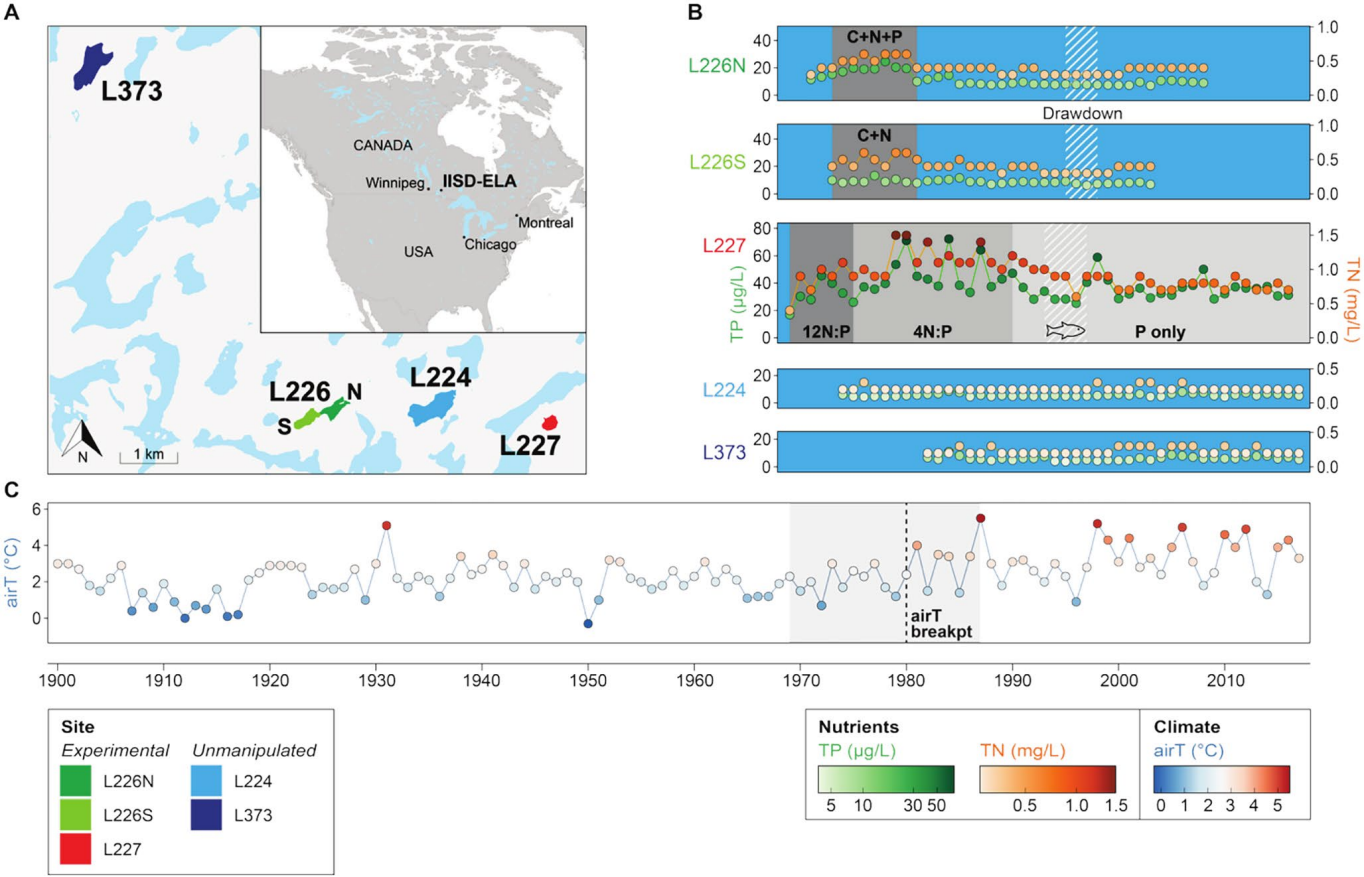
Overview of the study systems shown through (A) maps of the region and lakes within the ELA, (B) mean annual epilimnetic nutrient concentrations (TP in green; TN in orange; colour intensity indicates magnitude of nutrient concentration), and (C) mean annual air temperature trend (shown on a gradient of cool [blue] to warm [red]). Grey panels in nutrient plots show experimental regimes in L226 and L227 (blue panels indicate eras with no manipulation). The two‐basin nutrient loading experiment in L226 was performed between 1973 and 1980 and consisted of adding C, N, and P to the northwest basin (L226N) and C and N to the southwest basin (L226S). While both L226 basins had increases in algal biomass, P‐enriched L226N experienced much greater growth (Findlay and Kasian 1987). Water level drawdown was performed in L226 between 1995 and 1997; however, algal communities were mostly unaffected (Turner et al. 2005). L227 has been continuously fertilised at decreasing N:P since 1969. Nutrient loading regimes in L227 began with additions of 12 N:P by weight between 1969 and 1974, followed by 4 N:P between 1975 and 1989, and P only since 1990 (Schindler et al. 2008). Despite discontinued N additions in 1990, continued P additions, a large sediment P reservoir (O'Connell et al. 2020), and biological N fixation have sustained elevated algal biomass in L227 (Higgins et al. 2018). Northern pike were added into L227 in 1993 then removed in 1996 (indicated by a fish silhouette) for a trophic cascade experiment (Elser et al. 2000). Experimental regimes are detailed in the Experimental Procedures. L224 and L373 were unmanipulated. The air temperature breakpoint is indicated by a dashed vertical line at 1980, surrounded by 95% confidence limits indicated by grey panels. The x‐axis for the nutrient concentration plots (B panels) is shared with and located below the air temperature plot (C).

The experimentally fertilised sites (L226N, L226S, and L227; maximum depths 14.7, 11.6, and 10.0 m, respectively) and unmanipulated sites (L224 and L373; 27.3 and 21.2 m) are situated in the IISD‐ELA in northwestern Ontario, Canada (Figure 1). The two basins of L226 were divided by a plastic curtain between 1973 and 1985. Between 1973 and 1980, the southwest basin (L226S) was fertilised with C and N and the northeast basin (L226N) was fertilised with C, N, and P (Findlay and Kasian 1987). L226 was considered to have recovered to pre‐fertilisation water chemistry and algal composition within a few years following the end of the experimental nutrient loading (Turner et al. 2005). L227 has been subjected to ongoing nutrient loading since 1969. While experimental P loading has remained approximately constant since 1969, the ratios of N:P have decreased stoichiometrically over the years. An initial five‐year experiment (1969–1974) of loading N:P in a 12:1 ratio by weight was designed to test C limitation under N + P levels for balanced algal growth. In the following 14 years (1975–1989), N:P was loaded at 4:1 to test whether N:P values below the Redfield ratio select for N‐fixing Cyanobacteria to meet high cellular N demands. Since 1990, only P has been added to test the potential for managing algal blooms by restricting N loading (Higgins et al. 2018; Schindler et al. 2008).

A few other manipulations have also been conducted in the lakes. In L226, there were radioisotope tracer additions (1977–1978; 1989) and a water level drawdown experiment (1995–1997) (Turner et al. 2005). Prior to 1993, L227 had abundant minnow populations. Between 1993 and 1996, northern pike were added to test the effects of a trophic cascade on algal biomass (Elser et al. 2000). L227 has been fishless since 1997. The hypolimnion of L227 has likely been anoxic for at least 300 years predating experimentation, as evidenced by seasonal biogenic varves typically formed under bottom water anoxia (Wolfe et al. 1994). Although considered an unmanipulated Long‐Term Ecological Research site, L224 received metal radiotracer additions in 1976 (Hesslein et al. 1980).

### 2.2 Monitoring

Sampling for physical and chemical variables and phytoplankton occurred at the deepest part of each site, typically on a biweekly basis through the ice‐free season (Stainton et al. 1977; Havens et al. 2024). Water samples were collected from the epilimnion (surface to thermocline) and an operationally defined metalimnion (thermocline to depth of 1% surface irradiance) using an integrated sampler (Shearer 1978). 40‐mL subsamples of the unfiltered integrated water samples from the epilimnion and metalimnion were placed in a scintillation vial and preserved with 500 μL Lugol's solution. Total nitrogen (TN) and total phosphorus (TP) were calculated as the sum of the dissolved (TDN, TDP) and particulate (PN, PP) fractions after filtering the lake water through a 42.5 mm GF/C filter with a 1.2 μm nominal pore size. TDN and TDP were digested using sulfuric and hydrogen peroxide catalysed UV digestion. For TDN, nitrate and nitrite in the UV digest were converted to ammonia using a zinc column treated with dilute sulfuric acid followed by an indophenol blue colorimetric method. For TDP, the UV digest was analysed using the molybdenum blue colorimetric method. PN retained on a 25 mm GF/C filter was analysed using an Elemental Analyser (Exeter Analytical). For PP, particulate retained on the GF/C filter was baked at 500°C for 1.25 h followed by a dilute hydrochloric acid digestion (100°C for 2.2 h). The resulting orthophosphate was measured using the molybdenum blue colorimetric method on a spectrophotometer. Mean annual nutrient concentrations were calculated based on data that were linearly interpolated for missing spring, summer, and fall seasons across years (winter data were sparse and therefore excluded) for the years of available data (L226N 1971–2009; L226S 1973–2004; L227 1969–2018; L224 1974–2018; L373 1982–2018).

Algal biomass was monitored using morphological identification and enumeration by light microscopy (Findlay et al. 1994). 125‐mL subsamples of epilimnetic and metalimnetic water were preserved in Lugol's solution until analysis. 2‐mL aliquots of the subsamples were gravity‐settled for 24 h and species identification and enumeration were undertaken at 125× and 550× using the Utermöhl method. Cell measurements were undertaken to a maximum of 50 individuals within each species and cell numbers were converted to wet weight biomass by fitting shapes to geometric formulae assuming a specific gravity of 1.0. Algal monitoring was summarised for the years of available data (L226N 1971–2004; L226S 1973–2004; L227 1970–2018; L224 1974–2018; L373 1983–2018). Total annual spring–summer biomass in the epilimnion was calculated for each taxon, which was generally resolved to species. The monitoring taxonomy was harmonised to the Protist Ribosomal Reference database (PR^2^) v.5.0.0 (Guillou et al. 2012) to enable comparison with the molecular genetic data and to assign trophic functions according to the method described below. Taxa that were assigned to phototrophs or mixotrophs were retained as algae.

Historical daily mean air temperature data were accessed from Kenora (1900–1938) and Kenora A (1939–2018) weather stations (Environment and Climate Change Canada 2024). Missing daily data were filled in with the monthly average based on available days; missing monthly data were linearly interpolated between the same month over different years. Mean annual air temperature data from Kenora (A) stations were strongly correlated with air temperatures measured at the ELA L239 meteorological station for the years 1970–2017 (*r* = 0.97); therefore, Kenora (A) station air temperature data from 1900 onward were used in statistical analyses.

### 2.3 Sediment Sampling, Sectioning, and Dating

Sediment cores were collected between August 11–16, 2018, by the Natural Sciences and Engineering Research Council (NSERC) Canadian Lake Pulse Network (Huot et al. 2019). Sampling was conducted near the deepest point of each lake (NSERC Canadian Lake Pulse Network 2021) and sediments were collected using a National Lakes Assessment gravity corer (Aquatic Research Instruments) fitted with a 68 mm‐diameter core tube. The collected cores for all sites exceeded the 32‐cm length required to reach the preindustrial background for the Boreal Shield (NSERC Canadian Lake Pulse Network 2021) (L226N 42.5 cm; L226S 44.0 cm; L227 45.0 cm; L224 36.5 cm; L373 41.5 cm). Most of the overlying water was siphoned off from each core tube, and the remainder was jellified through the incremental addition of sodium polyacrylate (Zorbitrol). Core tubes were wrapped in black plastic bags to prevent light infiltration and shipped to Université Laval in Quebec City, Canada, where they were stored horizontally at 4°C.

Sediment cores were split into vertical halves while contained in the core tube. Age‐dating via gamma spectrometry measuring lead‐210, lead‐214, and caesium‐137 was performed on one core half as described in ref (Baud et al. 2023). Sediment age‐dates for each core were estimated using the constant rate of supply model (Baud et al. 2022) (Figure S1).

The second core halves were sectioned at 0.5‐cm intervals starting with the oldest sediments at the bottom. Sectioning was performed in a dedicated room using precautions to avoid contamination with modern DNA (Capo et al. 2021), including the use of sterile equipment. Subsamples for genetic analysis were collected from the centre of intervals into sterile 5‐mL tubes to avoid smeared or contaminated outer sediments in contact with the core tube. Subsamples were stored at −20°C.

### 2.4 DNA Extraction, Amplification, and Sequencing

DNA extraction from sediments was performed in a dedicated facility using NucleoSpin Soil kits (Macherey‐Nagel) with cell lysis SL1 and enhancer SX buffers. DNA was quantified in a Qubit 2.0 fluorometer (Thermo Fisher Scientific). A ~265 bp fragment of the 18S rRNA gene V7 region was PCR‐amplified using 960F (5′‐GGCTTAATTTGACTCAACRCG‐3′) (Gast et al. 2004) and NSR1438 (5′‐GGGCATCACAGACCTGTTAT‐3′) (van de Peer et al. 2000) primers, paired to target a broad diversity of microeukaryotes (Capo et al. 2016). Each 25 μL PCR mixture contained 14.25 μL Milli‐Q water, 5 μL High‐Fidelity buffer, 1.25 μL of each primer (10 μM), 0.5 μL dNTPs (10 mM), 0.25 μL dimethyl sulfoxide, 0.5 μL Phusion polymerase (Thermo Fisher Scientific), and 2 ng DNA. PCR conditions comprised a 1 min initial denaturation at 98°C, 30 cycles of 10 s at 98°C, 30 s at 60°C, and 20 s at 72°C, and a final 5 min extension at 72°C. PCR products were electrophoresed in 2% agarose gel for 100 min at 40 V then extracted at the target band length using QIAquick Gel Extraction kits (QIAGEN) modified by final elution into Milli‐Q. PCR products were submitted to Genome Quebec for library preparation and paired‐end sequencing (PE250) on an Illumina MiSeq.

### 2.5 Bioinformatic Analysis of 18S rRNA Amplicons

Primer sequences were trimmed from demultiplexed reads in Cutadapt v.3.1 (Martin 2011). The DADA2 v.1.16 pipeline was used to quality‐control reads, infer amplicon sequence variants (ASVs) across pooled samples, merge reads, remove chimeric contigs, and assign taxonomy (Callahan et al. 2016). Taxonomy was assigned with PR^2^ (Rodgers 2021) v.5.0.0 (Guillou et al. 2012). To further quality‐control the ASV dataset, highly divergent ASVs representing potentially spurious sequences were removed based on a de novo alignment performed in MAFFT (Katoh et al. 2019). Remaining primer sequences were trimmed based on the alignment of ASVs against the SILVA 99% non‐redundant small subunit rRNA database v.138.1 performed in SINA v.1.7.2 (Pruesse et al. 2012; Quast et al. 2012). ASVs assigned to Metazoa (animals) or Embryophyceae (land plants) were removed to retain a dataset of protists and fungi. Taxa were assigned to trophic functional groups based on nutritional modes reviewed by Adl et al. (2019) and FUNGuild, a database specific to fungi (Nguyen et al. 2016). ASVs of phototrophic and mixotrophic taxa were categorised as algae.

### 2.6 Statistical Analyses

Data analysis and visualisation were performed in R v.4.2.0. Multivariate correlations between algal monitoring and paleogenetic data in each lake were calculated using RV coefficients in FactoMineR v.2.3 (Lê et al. 2008). Assemblages were Hellinger‐transformed prior to computing RV coefficients. To compare annually resolved monitoring assemblages with multiyear‐integrated paleogenetic assemblages within overlapping time frames, we harmonised the datasets by calculating the mean algal biomass in monitoring assemblages within the estimated time frames captured in sediment intervals. Assemblage comparisons were made at the level of monitoring “taxon codes,” the finest level of taxonomic resolution by microscopy and often reflecting species assignments, against paleogenetic ASVs. Assemblage comparisons were subsequently made at the genus level for classified taxa. Taxa representing ≥ 1% of biomass or sequences were reported as prevalent in monitoring or paleogenetic records, respectively.

Principal component analyses (PCAs) of the taxonomic variation among annually resolved monitoring or multiyear‐integrated paleogenetic records for each site were performed on Hellinger‐transformed assemblages in vegan v.2.6.4 (Oksanen et al. 2022). To evaluate whether the first principal component (PC1) of each PCA reflected the chronology of algal records, relationships between PC1 and year were assessed using Pearson correlations. Breakpoints delineating climate periods in the mean annual air temperature time series were identified via structural change analysis in strucchange v.1.5.4; the number of breakpoints (1) was determined by selecting the model with the lowest BIC. Analysis of similarities (ANOSIM) was used to evaluate differences between monitoring or paleogenetic assemblages across unmanipulated conditions and nutrient loading regimes in each fertilised site and between climate periods (i.e., before and after the air temperature breakpoint year) in unmanipulated sites. Similarity percentages (SIMPER) analysis was used to calculate the mean contributions of taxa to algal community differences between the unmanipulated conditions and nutrient loading regimes in each fertilised site and between climate periods in unmanipulated sites; key taxa were identified up to a cumulative dissimilarity of 0.7 between assemblages. ANOSIM and SIMPER were each performed on Bray‐Curtis distances (i.e., percentage differences) of algal assemblages in vegan using 999 permutations.

Generalised additive models (GAMs) were constructed to assess nutrient and air temperature effects on algal community turnover in monitoring or paleogenetic records of each site. The response variable was the first principal component of algal community variation in individual sites. Because nutrient and air temperature data were available for different time frames (after 1969 for nutrients depending on the lake and since 1900 for climate), models were constrained by the years of available data. Therefore, GAMs using nutrient predictors leveraged data from the start of monitoring (1969 or after), whereas GAMs using air temperature only were based on data within the monitoring period or since 1900 to fit full paleogenetic records. To compare the contributions of nutrients and climate over a common time frame (after 1969), another set of GAMs was constructed based on nutrient and air temperature predictors. GAMs were constructed in mgcv v.1.8.31 (Wood 2019) using restricted maximum likelihood estimation for model smoothing. When the estimated degrees of freedom were within 0.5–1.5 for a particular variable, the GAM was recomputed with a linear smooth term (i.e., dropping the s() argument). Because smoothing functions are limited by the number of response data points, the number of knots used to smooth terms was iteratively decreased until convergence was reached; if decreasing the number of knots to 1 did not suffice to reach convergence, linear terms were used.

### 2.7 Code Availability

Scripts associated with this study are available at https://github.com/rebeccagarner/paleoELA.

## 3 Results

### 3.1 Synchrony of Monitoring and Sediment Records

**FIGURE 2:**
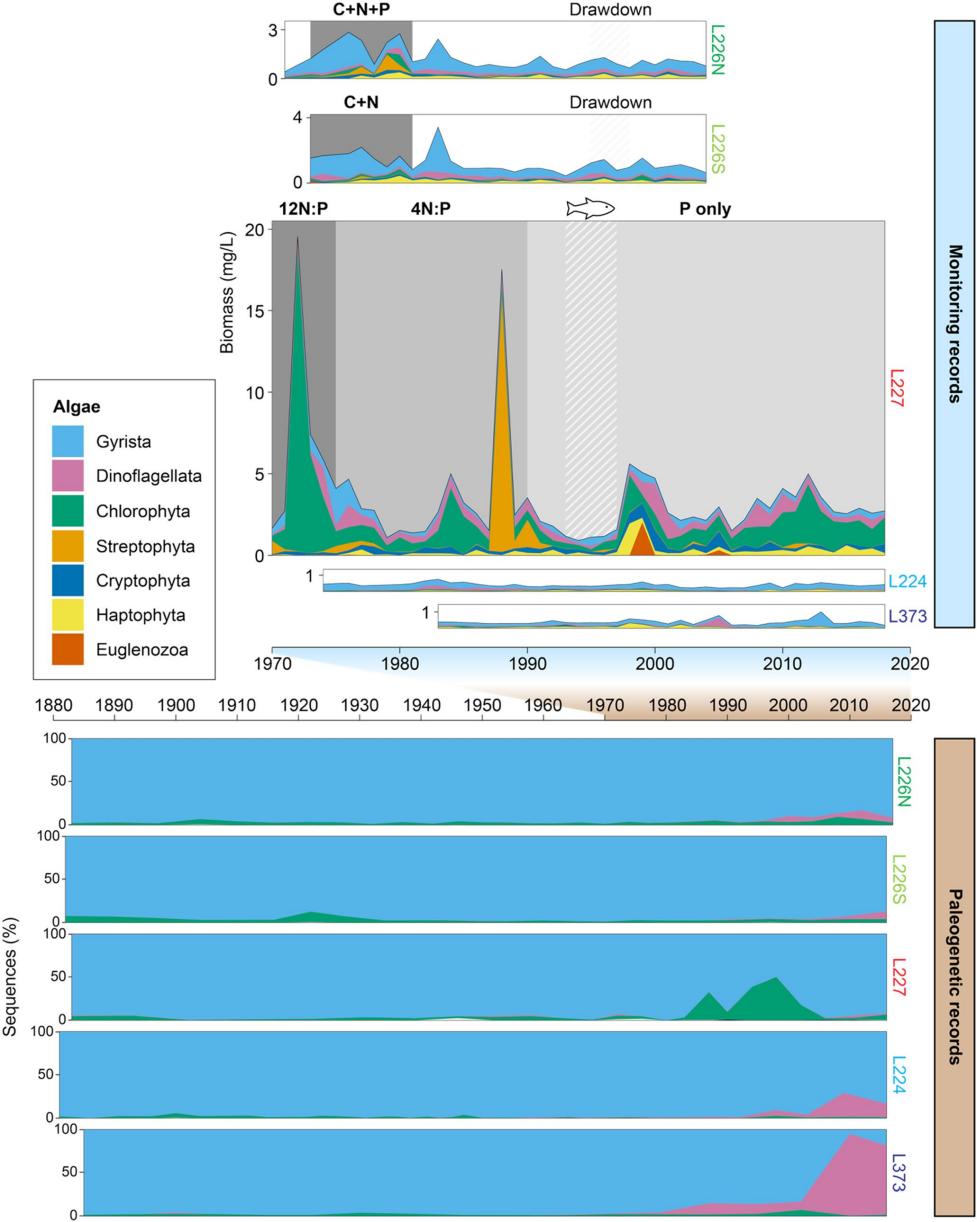
Algal diversity in experimental and unmanipulated lakes recorded in microscopy‐based monitoring (top) and paleogenetic (bottom) time series. Monitoring records show spring–summer algal biomass in the epilimnion. Paleogenetic records are presented as proportions of taxa occupying the algal assemblage sequence space for each sediment interval. The x‐axes for monitoring and paleogenetic records are positioned between the plots of each technique and highlight the overlap between the records (blue vs. brown). Grey panels in monitoring plots show experimental regimes in L226N, L226S, and L227 (described in Figure 1 caption).

Decades of traditional microscopy‐based monitoring in the ELA (Figure 1) have clearly demonstrated strong responses of eukaryotic algae and Cyanobacteria (which are not considered in our analyses) to nutrient loading (Findlay and Kasian 1987; Schindler et al. 2008) (Figure 2). L226N and L226S basins, which were differentially fertilised between 1973 and 1980 with C + N + P and C + N, respectively, experienced higher total spring–summer eukaryotic algal biomass during the experimental period, compared to the post‐experimental years with available monitoring data (1981–2004). On average, L226N supported greater biomass than L226S during the experimental period (L226N mean 2.045 mg/L; L226S mean 1.634 mg/L), whereas both basins contained lower biomass after nutrient additions ceased (L226N mean 1.019 mg/L; L226S mean 1.070 mg/L). Algal assemblages in the two sites were characterised by high chrysophyte biomass during the experimental era. L227, which was continuously fertilised under decreasing N:P regimes since 1969, experienced large variations in spring–summer algal biomass over 49 years of monitoring, averaging the highest biomass during the era of 12 N:P treatments (1969–1974; mean 7.440 mg/L) and lower biomass during the subsequent 4 N:P (1975–1989; mean 3.653 mg/L) and P‐only regimes (1990–2018; mean 2.783 mg/L). Algal assemblages in L227 were characterised by chlorophytes (e.g., *Oocystis* and *Ankistrodesmus*), dinoflagellates, and chrysophytes during the 12 N:P regime, streptophytes (e.g., *Spondylosium*), chlorophytes (e.g., *Pediastrum*), and dinoflagellates during the 4 N:P regime, and chlorophytes (e.g., *Dictyosphaerium*) and dinoflagellates during the P‐only regime. Although our analyses focus on eukaryotic microalgae, large increases in Cyanobacteria biomass notably occurred in L226N (Findlay and Kasian 1987) and L227 (Schindler et al. 2008). Unmanipulated lakes harboured low spring–summer eukaryotic algal biomass in most years (L224 range 0.211–0.748 mg/L; L373 0.171–1.003 mg/L).

Here, we extended the time series through a paleogenetic lens back across ~120 years of atmospheric warming (Figure 2). In all, 971 algal 18S rRNA ASVs encompassed 4,099,386 sequences (representing 65% of the total microeukaryotic dataset; Figure S2; Table S1) across 135 sediment intervals spanning the five cores. Monitoring and paleogenetic records are collected and measured on different timescales and capture different but overlapping algal assemblages. Whereas biweekly monitoring was conducted at a single limnetic location at targeted depths, sediments constantly integrate algal remains from across the lake and water column, including unmonitored littoral and benthic zones.

**FIGURE 3:**
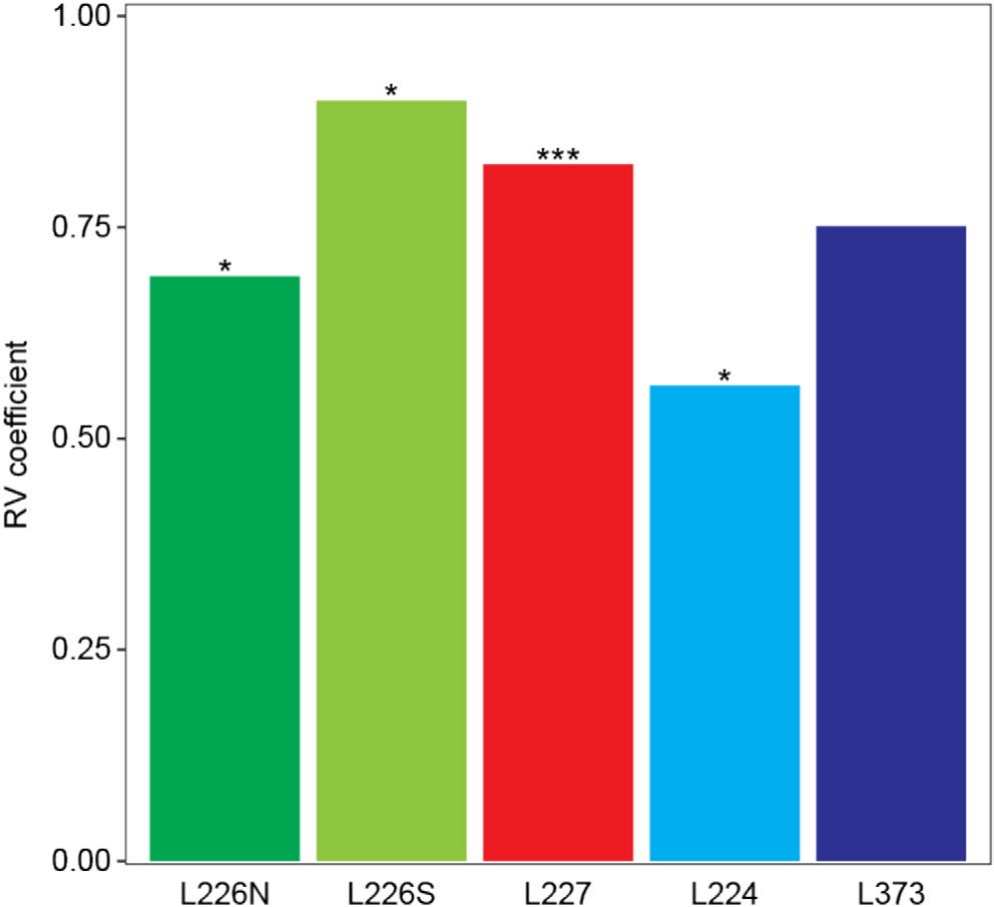
RV coefficients measuring the multivariate correlation between monitoring and paleogenetic algal community times series at the highest levels of taxonomic resolution for L226N (*n* = 10), L226S (*n* = 6), L227 (*n* = 12), L224 (*n* = 9), and L373 (*n* = 5). Asterisks indicate the level of statistical significance: *p* < 0.05 (*), *p* ≤ 0.001 (***).

Monitoring and paleogenetic time series for each site were harmonised to common time frames by averaging algal biomass in monitoring assemblages within the corresponding estimated depositional years of sediment intervals (Table S2). Contrasting the multiyear averaged biomass of microscopy‐identified taxa with sediment genotype compositions in harmonised datasets, we compared the temporal dynamics of algal assemblages in monitoring and paleogenetic records at the highest‐resolution taxonomy for each method. The two data streams were strongly correlated in L226N, L226S, L227, and L373 (RV multivariate correlation coefficient 0.69–0.90) and moderately correlated in L224 (RV coefficient 0.56) (Figure 3). When compared at the genus level, monitoring and paleogenetic datasets remained moderately to strongly correlated across sites (RV coefficient 0.55–0.85; Figure S3). The strength of these correlations despite the differences between techniques is likely due to the multiyear smoothing of noise sampled by the more sharply resolved monitoring time series. The correlations of monitoring and paleogenetic records in our analysis exceed comparisons of microeukaryotic assemblages recovered from water column sampling and sediment traps over a three‐year study in Cultus Lake, located in western Canada (Gauthier et al. 2021). Temporal community dynamics exposed by monitoring and paleogenetics appear to converge over longer time frames (Monchamp et al. 2016; Thorpe et al. 2022, 2023; Mejbel et al. 2023). The strong congruence between the multidecadal monitoring and paleogenetic records of algal communities in multiple lakes represents a critical validation step in the analysis of sediment DNA to reconstruct historical ecosystem dynamics.

Despite the congruence in temporal community changes, there were taxonomic differences between techniques (Figure 2). Of the major algal groups identified by microscopy‐based monitoring, Gyrista (chrysophytes and diatoms), dinoflagellates, and chlorophytes left abundant DNA traces in sediments (achieving as high as 87%, 70%, and 94% of annual biomass and 100%, 94%, and 50% of sequences per sediment interval across sites, respectively). Monitoring and paleogenetic time series correlations for these lineages across lakes were on average highest for Gyrista (RV coefficient 0.52–0.87) and lowest for dinoflagellates (RV coefficient 0.26–0.63; Figure S4), aligning with previously reported rank order of time series coherence for algal phyla (Thorpe et al. 2023). Streptophytes, haptophytes, and cryptophytes were prevalent in monitoring yet rare in sediments. The underrepresentation of cryptophytes in other paleogenetic studies applying the same and other primer sets has been attributed to limited DNA deposition in sediments due to zooplankton grazing and the lack of a protective cell wall, which may make the alga more vulnerable to rupture (Gauthier et al. 2021; Thorpe et al. 2023; Capo et al. 2015). Despite some DNA diagenesis, paleogenetics is not limited by morphological preservation (Domaizon et al. 2017) and detected an expanded diversity of microeukaryotes including apicomplexans, fungi, cercozoans, ciliates, and many other groups at lower sequence abundances (Figure S2; Table S1). Compared with microscopy‐based monitoring, paleogenetics achieves far broader and higher resolution taxonomic scope through DNA analysis and an integration of species from across lake habitats, as observed by the detection of algae associated with littoral or benthic zones (e.g., *Staurosira*, *Eunotia*, *Staurastrum*, and *Sellaphora* spp.).

### 3.2 Algal Community Shifts Across Nutrient Loading Regimes and Climate Periods

**FIGURE 4:**
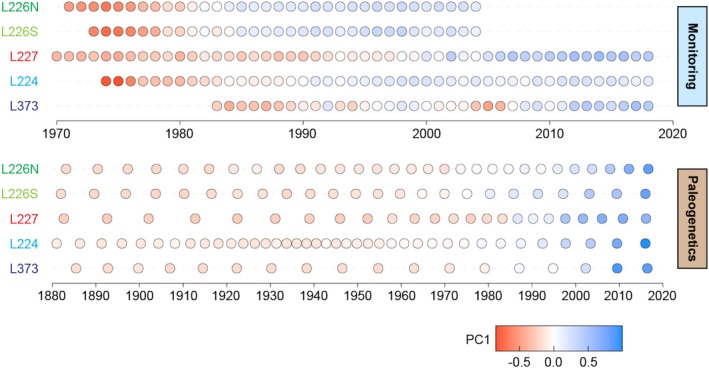
Chronologies of monitoring (top) and paleogenetic (bottom) records of each site with point colours indicating the coordinates of algal assemblages on the primary axis of taxonomic variation (PC1). A separate principal component analysis of algal taxonomic variation was performed for each lake and technique.

To assess changes in algal community composition across full time series, PCAs were conducted on annually resolved monitoring or multiyear‐integrated paleogenetic assemblages in each site (Figures S5, S6). In each PCA, the primary axis (PC1) explained a substantial proportion of the total variance in algal community composition (monitoring 20%–30%; paleogenetics 43%–66%). Moreover, PC1 clearly reflected chronological shifts in algal assemblages as evaluated by strong correlations between PC1 and time for each method (monitoring *r* = 0.74–0.93; paleogenetics 0.72–0.83; Figure 4; Table S3).

Nutrient loading regimes marked significant algal community shifts in experimental lakes (ANOSIM *p* < 0.001). L226N and L226S were partitioned for the experimental nutrient loading and recovered to an oligotrophic state after the seven‐year experiments ceased in 1980. Strong differences between fertilisation‐ and recovery‐era monitoring assemblages were detected by ANOSIM in L226N (*R* = 0.89) and L226S (*R* = 0.79) (Figures S5, S6; Table S4). L227 monitoring assemblages changed moderately (*R* = 0.50) between 12 N:P, 4 N:P, and P‐only experimental regimes. Monitoring in L226 and L227 was initiated close to the start of nutrient addition experiments; therefore, pre‐manipulation monitoring records are not available for comparison. Substantial pre‐manipulation records were supplied by paleogenetic time series, which reconstructed algal assemblages back to *circa* 1880 (Figure 2). Paleogenetic records reflected moderate to strong differences between algal assemblages across pre‐manipulation, fertilisation, and recovery eras, where relevant (L226N *R* = 0.57; L226S *R* = 0.66; L227 *R* = 0.51). Structural change analysis of the ~120‐year air temperature data identified 1980 as the year marking the climate warming breakpoint. In the unmanipulated lakes, significant algal community shifts were detected across the 1980 climate warming breakpoint, with stronger changes in the paleogenetic records (L224 *R* = 0.77; L373 *R* = 0.89) than in the monitoring (L224 *R* = 0.57; L373 pre‐1980 monitoring records are not available) (Figures S5, S6; Table S4).

**FIGURE 5:**
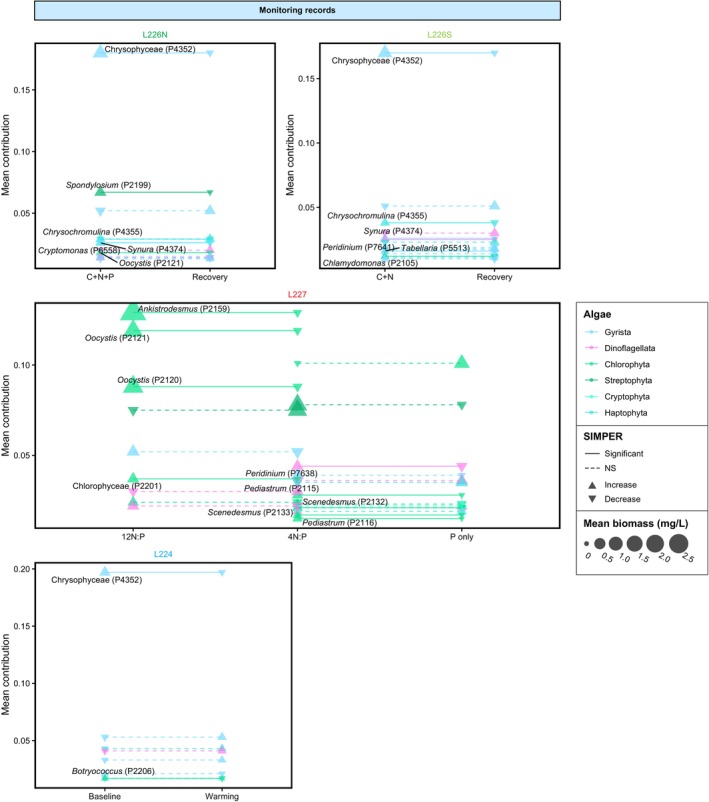
Mean contributions of key taxa identified by SIMPER analysis contributing to the differences in monitoring assemblages between specific nutrient loading regimes, recovery, and warming in each site. Labels and solid lines identify taxa with statistically significant contributions to the dissimilarity between assemblages (SIMPER *p* < 0.05); non‐significant taxa are identified by dashed lines. Point colours indicate the taxonomic subdivision and point sizes represent taxon mean biomass in a set of assemblages. Point shapes indicate whether a taxon increases (up arrow) or decreases (down arrow) on average across periods.

**FIGURE 6:**
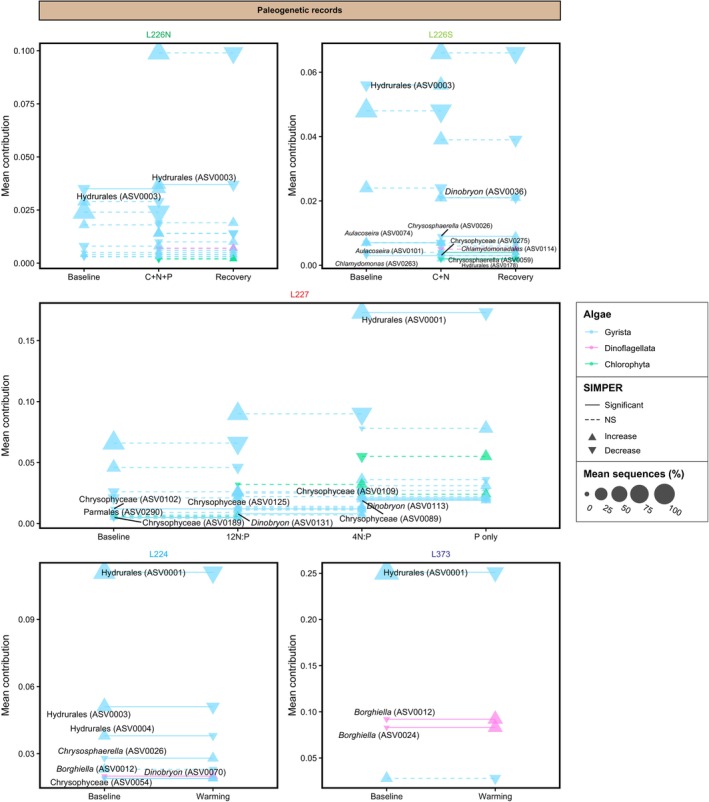
Mean contributions of key taxa identified by SIMPER analysis contributing to the differences in paleogenetic assemblages between baseline/pre‐manipulation conditions, specific nutrient loading regimes, recovery, and warming in each site. Labels and solid lines identify taxa with statistically significant contributions to the dissimilarity between assemblages (SIMPER *p* < 0.05); non‐significant taxa are identified by dashed lines. Point colours indicate the taxonomic subdivision and point sizes represent taxon mean sequence composition in a set of assemblages. Point shapes indicate whether a taxon increases (up arrow) or decreases (down arrow) on average across periods.

SIMPER analysis highlighted key species contributing to algal community shifts across conditions (Figures 5, 6; Table S5). Transitions from pre‐manipulation (i.e., baseline) to nutrient loading conditions in all experimental sites (L226N, L226S, and L227) were identified exclusively from paleogenetic records and characterised by fluctuations in chrysophytes of the order Hydrurales. Three Hydrurales genotypes were consistently the most sensitive to nutrient additions across sites, including one ASV that increased in relative composition between baseline and experimental conditions, and two ASVs of a different family that decreased (the directionality of taxon changes between conditions are displayed in Figure 6). In L226S, *Aulacoseira* diatoms were more prevalent during C + N additions. For time frames when both sets of records were available, monitoring and paleogenetic time series distinguished different species, due at least in part to differences in analytical methods and taxonomic scope. In L227, comparison of monitoring records between 12 N:P and 4 N:P regimes showed decreases in chlorophytes (*Ankistrodesmus*, *Oocystis*, and unclassified sp.) and between 4 N:P and P‐only treatments, decreases in dinoflagellate *Peridinium* and chlorophytes of the order Sphaeropleales (*Pediastrum* and *Scenedesmus*). In paleogenetic records, decreases in Hydrurales and increases in chlorophyte *Desmodesmus* were key to transitions between all L227 nutrient loading regimes. Chrysophytes *Dinobryon* and *Cyclonexis* in L227 were also sensitive to changes, increasing from 12 N:P to 4 N:P and 4 N:P to P‐only treatments, respectively.

During the recovery conditions (exclusive to L226), some taxon overlap was evident between the L226N and L226S basins. In L226N monitoring records, the recovery from the C + N + P experiment was characterised by decreases in an array of groups including chrysophytes (unclassified and *Synura*), streptophytes (*Spondylosium*), haptophytes (*Chrysochromulina*), cryptophytes (*Cryptomonas*), and chlorophytes (*Oocystis*). Echoing some of the same species as the northeast basin, the L226S recovery from the C + N experiment was characterised by decreases in chrysophytes (unclassified and *Synura*), haptophytes (*Chrysochromulina*), dinoflagellates (*Peridinium*), diatoms (*Tabellaria*), and chlorophytes (*Chlamydomonas*). *Chlamydomonas* was indicative of the L226 recovery in both the monitoring and paleogenetics records. Paleogenetics consistently identified decreases in Hydrurales as key to differentiating fertilisation‐ and recovery‐era assemblages in both L226N and L226S, while increases in chrysophytes (*Dinobryon* and *Chrysosphaerella*) and chlorophytes (*Chlamydomonas*) additionally characterised the recovery in L226S.

In unmanipulated L224, transitions from baseline conditions across the 1980 warming breakpoint were marked by decreases in chrysophytes in the monitoring record (pre‐1980 monitoring records are not available for L373). The longer paleogenetic records showed changes in chrysophytes (decreases in Hydrurales and increases in *Chrysosphaerella* and *Dinobryon*) in L224 and changes in Hydrurales (decreases) and dinoflagellates (increases in *Borghiella tenuissima*) in L373. In each of the unmanipulated sites, mixotrophic chrysophytes and dinoflagellates were indicative of warming, aligning with climate‐driven community shifts previously identified by monitoring (Findlay et al. 2001). The increase in mixotrophs was previously attributed to drought conditions in the 1980s which reduced terrestrial runoff and produced low‐nutrient water columns with deeper light penetration that may have advantaged mixotrophic species freed from light limitation and capable of exploiting bacterial prey in the deeper, nutrient‐rich waters. The presence and sensitivity of Hydrurales in all sites, regardless of conditions, suggest indicator species potential and a level of shared community baseline. However, as shown in the PCAs conducted on monitoring or paleogenetic records among sites, trophic state clearly distinguished the assemblages in unmanipulated L224 and L373, and even the recovered L226N and L226S, from continually manipulated L227 (Figures S5, S6).

### 3.3 Effects of Nutrient and Climate Drivers on Algal Communities

Building on the observed algal community shifts and indicator species associated with experimental fertilisation and climate warming, we next quantified algal community responses to eutrophication and climate along nutrient and air temperature gradients in sites subjected to experimental nutrient loading (L226N, L226S, and L227) or no manipulation (L224 and L373). To this end, we constructed GAMs fitting algal community composition in the monitoring or paleogenetic records of each site to (1) nutrients (TP and TN), (2) climate (air temperature), or (3) nutrients and climate combined. Since the primary axis of variation in the PCAs captured chronological shifts in algal community composition (Figure 4), PC1 was input as the GAM response variable for each site.

**FIGURE 7:**
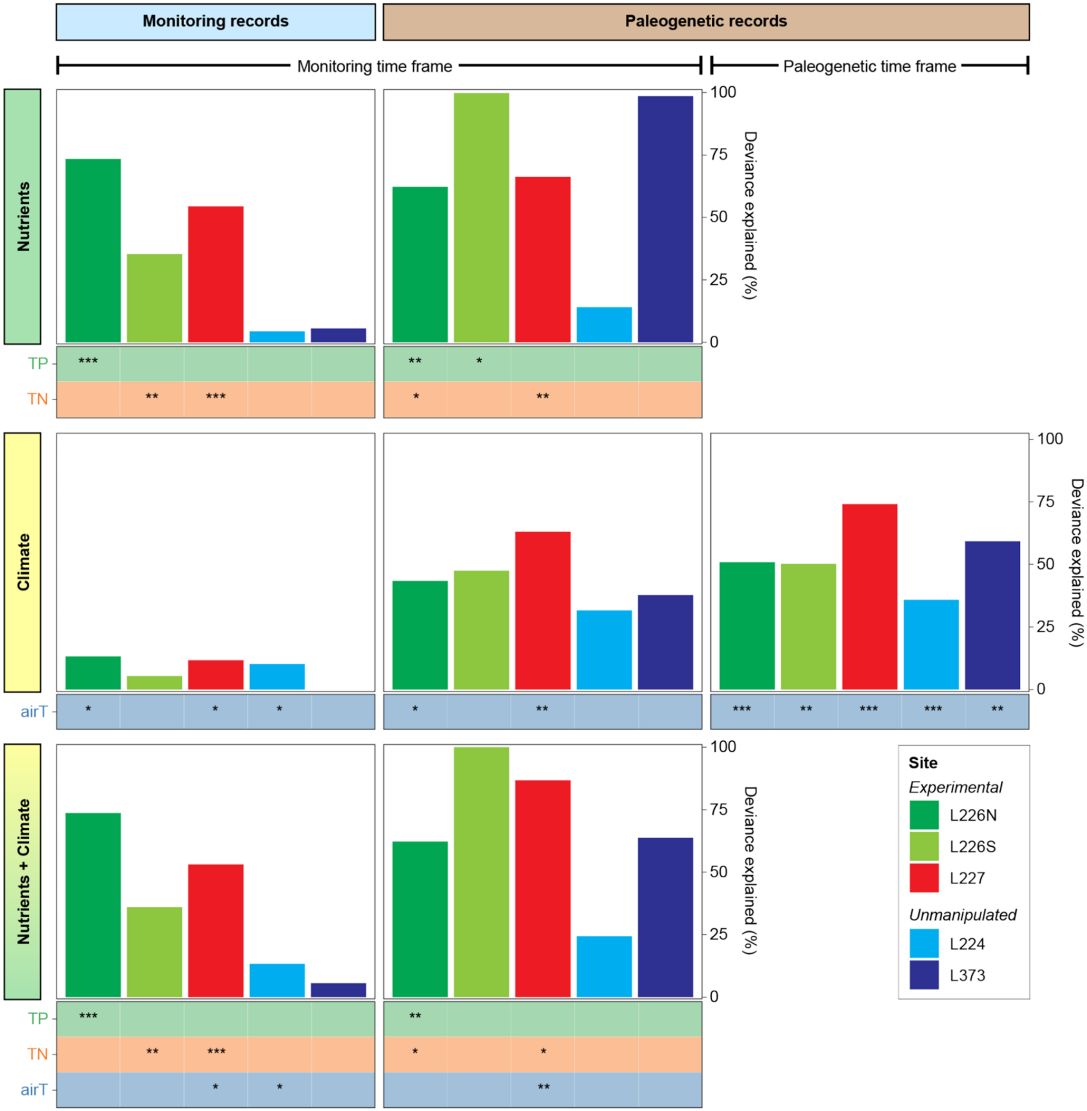
Deviance explained by GAMs fitting the variation in algal monitoring or paleogenetic records to nutrient (TP and TN), climate (air temperature), or additive nutrient and climate predictors. The responses of paleogenetic records were evaluated over the same time frames as monitoring records (from 1969 or later) to compare signal detection between techniques. The responses of paleogenetic records to 120 years of available air temperature data were also evaluated to assess climate signals over a longer perspective. Asterisks indicate the level of statistical significance of nutrient and/or climate predictors: *p* < 0.05 (*), *p* ≤ 0.01 (**), *p* ≤ 0.001 (***). Also see Table S6.

As expected, nutrients were strong and statistically significant drivers of algal community change in lakes with histories of experimental fertilisation. GAMs fitting the responses of monitored assemblages to nutrients alone explained moderate to high deviance in fertilised sites (deviance 35%–73%) and low deviance in unmanipulated lakes (4%–6%) (Figure 7; modelled effects are displayed in Figure S7). Responses were even stronger for paleogenetic assemblages in sediment intervals corresponding to monitoring periods, with nutrients explaining 62%–100% deviance in fertilised sites (Figure 7; Figure S8). The GAM for unmanipulated L373 paleogenetic records also explained high deviance (99%) despite containing no statistically significant nutrient variables, pointing to model overfitting of the limited response data or reliance on the intercept term. As expected, the longer paleogenetic records in unmanipulated L224 were not well explained by nutrient variation (deviance 14%). Nutrient variable significance (*p* < 0.05) was exclusive to models for fertilised sites and aligned with the monitoring data available for the experiments. For both monitoring and paleogenetic time series, TP was the leading driver of algal community change in L226N, reflecting its role as the limiting nutrient in the C, N, and P addition experiment. TN was the significant nutrient driver of algal community change in L227, reflecting the effect of the declining N:P ratios on algal assemblages and the discontinuation and replacement of experimental N loads by biological N fixation (Higgins et al. 2018). However, while constant P loads have clearly increased the trophic state of L227, the lack of pre‐manipulation data do not introduce sufficient TP variation for the models to be sensitive to its effects on algal communities in this system.

In GAMs fitting algal community responses to climate alone, modest effects of air temperature were detected in the monitoring records of some sites (L226N, L227, and L224; deviance 10%–13%) (Figure 7; Figure S7). Within the corresponding monitoring periods, the partial paleogenetic records of two fertilised systems (L226N and L227) reflected large and statistically significant increases in climate effects (deviance 43%–63%) over the monitoring records (Figure 7; Figure S8). Strikingly, when paleogenetic records were extended to fit the ~120‐year atmospheric warming trend, significant climate signals were detected in all sites, regardless of trophic state history (deviance 36%–74%) (Figure 7; Figure S8). The climate effects in the full paleogenetic records represented notable increases (7–11 percentage points) over the partial records of L226N and L227 within the monitoring periods. The strongest effect of climate on paleogenetic algal assemblages was observed in L227, the lake subjected to the longest and ongoing experimental fertilisation.

To evaluate potential synergies between eutrophication and climate, we constructed a final set of GAMs combining nutrient and air temperature predictors, restricting the time series to the window with available nutrient monitoring data (Figure 7; Figures S7, S8). The paleogenetic record from L227 showed a large increase in explained deviance when both drivers were combined (87%), relative to the separate nutrient (66%) and climate (63%) GAMs. In contrast, more parsimonious models could not be constructed from additive nutrient and climate terms for any other site based on monitoring or paleogenetic records. These findings reinforce that warming was not a major driver of algal community change over the monitoring period, unless the lake experienced prolonged eutrophication.

## 4 Discussion

In this study, we examined the long‐term effects of eutrophication and climate warming on algal communities through the analysis of rare multidecadal monitoring records and paleogenetic reconstructions. To evaluate algal community responses to nutrient and climate stressors, we investigated whole‐lake fertilisation experiments in the ELA, focusing on a continuously fertilised lake (L227) and a recovered two‐basin system (L226N and L226S) split by different nutrient treatments, and two unmanipulated lakes (L224 and L373) exposed only to a changing climate.

Despite differences in sampling strategy, analytical methods, and taxonomic scope, algal community dynamics in paleogenetic records were synchronised with contemporaneous monitoring. The demonstration that sediments archive the patterns of algal community turnover represents a key contribution in further validating the analysis of sediment DNA as a proxy for past lake ecosystems. Nonetheless, the preservation of historical community dynamics in paleogenetic records should be further investigated, given the taxonomic biases introduced by DNA taphonomy (Thorpe et al. 2023). We observed that some major algal groups were not well preserved, likely due to the degradation of DNA released from vulnerable cells and low deposition in sediment (Capo et al. 2015). A previous sediment core study in the ELA compared Cyanobacteria diversity identified by monitoring and paleogenetics (Mejbel et al. 2023), and we build on this with eukaryotic microalgae. We found that the congruence of monitoring and paleogenetic records varied among algal groups and that Gyrista dynamics were among the best preserved: results that align with findings from an earlier study conducted in Esthwaite Water (Thorpe et al. 2023). Overall, the preservation of community dynamics now demonstrated through a strong coherence of paleogenetic and monitoring records in multiple lakes gives us confidence to extend time series retrospectively to access pre‐monitoring and pre‐manipulation assemblages.

We clearly demonstrated the differential signal strengths of algal community drivers detected between monitoring and paleogenetic datasets. Paleogenetic records generally showed stronger effects of nutrients and air temperature than monitoring over the same time frames. Stressor detection in paleogenetic records is linked to the spatio‐temporal integration of algal communities in lake sediments. First, the integration of algal diversity from unmonitored habitats creates a spatially comprehensive record of the lake community incorporating species reactive to stressors (Barouillet et al. 2023). In particular, paleogenetic assemblages contained littoral species likely more sensitive to changes in temperature conditions and benthic species potentially impacted by climate‐driven alterations to the oxycline or nutrient resuspensions from sediments. Second, paleogenetic records compile stronger signals for eutrophication and warming as sediments integrate algal remains throughout the year. Annual air temperature increases in the ELA have primarily occurred in the fall and winter (Guzzo and Blanchfield 2017), outside the focal monitoring seasons. So, while monitoring is highly useful for extracting fine‐grained algal community dynamics not typically captured by sediment cores, paleogenetic records reflect algal community variation across habitats, seasons, and years, integrating where and when stressors are most influential.

Algal communities in fertilised sites responded to nutrients and air temperature over different time scales. Specifically, eutrophication was a fast driver of algal community change while climate was a slow driver (Carpenter et al. 2001). In the monitoring time series, nutrient effects were strong in fertilised sites, where air temperature effects were detected but relatively weak over a scale of a few decades. Moreover, the full effects of nutrient treatments on algal communities were not evident in the monitoring data because the lack of early records prevents the time series from being anchored in the oligotrophic pre‐manipulation states. Modelling the responses of paleogenetic assemblages to air temperature within the monitoring time frames allowed us to uncouple the effect of time series length. Paleogenetic assemblages in two fertilised sites showed air temperature effects within this truncated period. Yet, climate signals increased and were observed in all lakes when assessed over the 120‐year paleogenetic time series. Clearly, to detect the effects of slow drivers, lake ecosystems must be studied on long time scales rarely achieved by monitoring programs, but accessible through paleolimnology (Smol 2019; Davidson and Jeppesen 2013).

We detected an additive response to eutrophication and climate warming in paleogenetic records that was not evident in the monitoring. This combined effect was observed in L227, which was continuously fertilised for nearly five decades, and suggests that the food web was primed by the destabilising eutrophic conditions to respond to the subsequent stress of warming temperatures. In other words, the algal community underwent greater change in response to climate change because it was first destabilised by eutrophication. While spring–summer temperatures did not significantly increase over the monitoring period, the climate change effect is likely related to rising fall–winter temperatures promoting longer growing seasons annually (Salk et al. 2022). In the P‐only addition phase of the L227 fertilisation experiment, a first seasonal bloom of Cyanobacteria occurred annually in the summer (Higgins et al. 2018). The increases in fall temperatures appeared to lead to a greater chance and longer persistence of a second more diverse seasonal bloom of Cyanobacteria and chlorophytes, fuelled by nutrients recycled from the collapse of the first bloom (Higgins et al. 2018). Warming lake temperatures also increase algal metabolism and growth rates. Mechanistic modelling has shown that earlier and larger algal blooms were triggered in L227 as an effect of the interaction of climate change and fertilisation, compared with scenarios of fertilisation or climate change only (Salk et al. 2022).

While the current study considered nutrient and air temperature drivers, algal communities may also have responded to other variables. In particular, climate change alters lake ecosystems not only through rising air temperatures but through in‐lake and watershed‐scale processes (Jeppesen et al. 2014; Woolway et al. 2022). Precipitation is the main control on dissolved organic carbon (DOC) levels in ELA lakes (Imtiazy et al. 2020), while a warming climate has increased the runoff season (Meyer‐Jacob et al. 2019). Increased DOC has altered the physical properties of the unmanipulated lakes by reducing water clarity and shortening the thermoclines and euphotic zone depths, and thus decreasing depth‐integrated algal biomass (Sherbo et al. 2023). The ELA has experienced a ~31‐year precipitation cycle of wet and dry years, with the most recent drought through the 1970–80s (Parker et al. 2009). However, since the early 1990s, a wet period has persisted and precipitation has driven increased DOC loads in ELA lakes. Whether the interruption of the wet‐dry cycle is a consequence of climate change or is a clue that the cycle was a temporary phenomenon within a longer weather history, increased precipitation is a symptom of climate change in the region (Imtiazy et al. 2020; Emmerton et al. 2019). Our models of algal community change in unmanipulated L224 and L373 may be recapitulating some effects of precipitation and DOC, as no trophic state interaction would explain a climate response over the monitoring period. However, while a previous study of these lakes traced a proliferation of mixotrophic chrysophytes and dinoflagellates in monitoring assemblages to the drought period of decreased DOC (Findlay et al. 2001), our paleogenetic reconstructions appear to align these community shifts to the most recent wet period of increased DOC (Meyer‐Jacob et al. 2019).

Our study has shown the advantages of a paleogenetic perspective on long‐term algal community dynamics. However, the paleogenetic time series contained limitations. Foremost, sediment intervals corresponded to multiple and variable depositional years. While this was useful for integrating algal diversity and thus strengthening stressor signals, fewer data points existed within the paleogenetic time series truncated to monitoring periods. Consequently, GAMs tended to overfit sparse paleogenetic records as in L373, the most compressed sediment chronology. As such, it may be more challenging to extract coherent driver signals from lakes with low sedimentation rates or greater sediment mixing because temporal resolution will be lower. Furthermore, paleogenetic records were extracted from complex sediment DNA pools representing a broad microeukaryotic diversity, which detracts from amplifying historical algal signals. The sediment assemblages of the experimentally fertilised sites contained high sequence abundances of fungi, apicomplexans, and other parasitic alveolates which may inhabit the sediment subsurface and that were not considered part of the paleogenetic record. Many of these groups are associated with microaerophilic or anoxic environments (Adl et al. 2019) and are likely to thrive in oxygen‐depleted sediments often characteristic of eutrophic lakes. The prevalence of fungi and alveolates in sediments deposited after L226 recovered to oligotrophic status suggests that the sediment community may have been altered long‐term by eutrophication. High occurrences of these groups in the sediment column could be an indicator for past eutrophication, even for recovered lakes.

In conclusion, synchronised monitoring and paleogenetic time series showed strong responses of algal communities to eutrophication within recent periods of experimental nutrient loading. However, compared with the paleogenetic time series, monitoring appeared to underestimate the impacts of climate warming and its combined effects with eutrophication. As freshwater eutrophication is exacerbated on a warming planet, sediment DNA archives integrating lake diversity across habitats and seasons provide a retrospective detection system for slow‐acting stressors and their synergies.

## Author Contributions

**Rebecca E. Garner:** conceptualization, methodology, investigation, formal analysis, writing – original draft, writing – review and editing, funding acquisition. **Zofia E. Taranu:** methodology, formal analysis, writing – original draft, writing – review and editing. **Scott N. Higgins:** investigation, data curation, writing – original draft, writing – review and editing. **Michael J. Paterson:** investigation, data curation, writing – original draft, writing – review and editing. **Irene Gregory‐Eaves:** conceptualization, methodology, writing – original draft, writing – review and editing, funding acquisition. **David A. Walsh:** conceptualization, methodology, writing – original draft, writing – review and editing, funding acquisition.

## Conflicts of Interest

The authors declare no conflicts of interest.

## Supporting information


**FIGURE S1.** Sediment dating estimated using the constant rate of supply model. Sediment interval midpoint depths are along the x‐axis. Vertical bars indicate model error margins. Blue curves show model‐interpolated sediment age‐dates.
**FIGURE S2.** Taxonomic composition of microeukaryotic assemblages in 0.5‐cm sediment core intervals of experimental and unmanipulated sites, extending to the *circa* 1880 preindustrial background for the region. In total, 3953 18S rRNA gene amplicon sequence variants (ASVs) encompassing 6,345,459 sequences were inferred across 135 sediment samples spanning the five cores. Gyrista was by far the most abundant eukaryotic subdivision in terms of total sequences (61%) and ASVs (754) across samples. Each sediment layer contained 1645–54,473 sequences assigned to Chrysophyceae, comprising 2%–92% of sequences in each sample. Trophic functions were assigned to 76% of ASVs representing 95% of sequences. Of these, 971 ASVs encompassing 4,099,386 sequences (65% of the dataset) were assigned to algae (Figure 2), whose sunlight‐dependent nutrition distinguishes them as preserved groups unlikely to grow in the sediment column in situ. Dinoflagellates and other mixotrophs were abundant in top sediment layers. Apicomplexa (13% of sequences) followed by Fungi (10%) were the second and third most abundant subdivisions, particularly in the sediments of fertilised sites.
**FIGURE S3.** RV coefficients measuring the multivariate correlation between monitoring and paleogenetic algal community times series at the level of genera for L226N (*n* = 10), L226S (*n* = 6), L227 (*n* = 12), L224 (*n* = 9), and L373 (*n* = 5). Asterisks indicate the level of statistical significance: *p* < 0.05 (*), *p* ≤ 0.01 (**).
**FIGURE S4.** RV coefficients measuring the multivariate correlation between monitoring and paleogenetic records for prevalent algal subdivisions for L226N (*n* = 10), L226S (*n* = 6), L227 (*n* = 12), L224 (*n* = 9), and L373 (*n* = 5). Assemblages were compared at the highest levels of taxonomic resolution. Asterisks indicate the level of statistical significance: *p* < 0.05 (*), *p* ≤ 0.01 (**), *p* ≤ 0.001 (***).
**FIGURE S5.** Principal component analyses conducted on monitoring assemblages in individual (all except bottom‐right plot) and combined (bottom‐right plot) sites. Point labels and sizes indicate the monitoring year. Point colours indicate the site. Contour colours indicate specific nutrient loading regimes, recovery, and warming in each site.
**FIGURE S6.** Principal component analyses conducted on paleogenetic assemblages in individual (all except bottom‐right plot) and combined (bottom‐right plot) sites. Point labels and sizes indicate the sediment interval midpoint estimated year. Point colours indicate the site. Contour colours indicate baseline/pre‐manipulation conditions, specific nutrient loading regimes, recovery, and warming in each site.
**FIGURE S7.** Partial effects over time of individual predictors (TP in green; TN in orange; air temperature in blue to red gradient) on the taxonomic variation in monitoring assemblages fitted in GAMs. Grey panels show experimental regimes in L226N, L226S. and L227 (blue panels indicate eras with no manipulation).
**FIGURE S8.** Partial effects over time of individual predictors (TP in green; TN in orange; air temperature in blue to red gradient) on the taxonomic variation in paleogenetic assemblages fitted in GAMs. Separate contribution plots are displayed for GAMs based on paleogenetic records within monitoring or paleogenetic time frames. Grey panels show experimental regimes in L226N, L226S, and L227 (blue panels indicate eras with no manipulation).
**TABLE S2.** Monitoring time series harmonisation within corresponding sediment intervals. “*n*” is the number of data points.
**TABLE S3.** Summary of Pearson correlations between the primary axis of variation (PC1) and time for monitoring or paleogenetic records in each site.
**TABLE S4.** Summary of ANOSIM evaluating the global differences in monitoring or paleogenetic assemblages between all periods (selected from baseline/pre‐manipulation, specific nutrient loading regime, recovery, and warming) in each site.
**TABLE S6.** Summary of generalised additive model parameters and results. Nonlinear terms are indicated by s(). “*n*” is the number of data points.

**TABLE S1.** Amplicon sequence variant (ASV) composition of sediment intervals across sites. Includes the taxonomic and functional assignments of ASVs.

**TABLE S5.** Summary of SIMPER analysis identifying key taxa contributing to the differences in monitoring or paleogenetic assemblages between sequential periods (selected from baseline/pre‐manipulation, specific nutrient loading regime, recovery, and warming) in each site.

## Data Availability

Reads were deposited at the European Nucleotide Archive under study accession PRJEB47328 (https://www.ebi.ac.uk/ena/browser/view/PRJEB47328). Monitoring data are available upon request from the IISD‐ELA (https://www.iisd.org/ela/science‐data/our‐data/data‐requests/).
