# Supplementary material for: Eutrophication and Warming Drive Algal Community Shifts in Synchronised Time Series of Experimental Lakes
Source: Environ Microbiol. 2025 Jul 24;27(7):e70159. doi: 10.1111/1462-2920.70159 (PMC12288632; doi:10.1111/1462-2920.70159)
Supplement: Supplementary file 1 — FIGURE S1. Sediment dating estimated using the constant rate of supply model. Sediment interval midpoint depths are along the x‐axis. Vertical bars indicate model error margins. Blue curves show model‐interpolated sediment age‐dates. FIGURE S2. Taxonomic composition of microeukaryotic assemblages in 0.5‐cm sediment core intervals of experimental and unmanipulated sites, extending to the circa 1880 preindustrial background for the region. In total, 3953 18S rRNA gene amplicon sequence variants (ASVs) encompassing 6,345,459 sequences were inferred across 135 sediment samples spanning the five cores. Gyrista was by far the most abundant eukaryotic subdivision in terms of total sequences (61%) and ASVs (754) across samples. Each sediment layer contained 1645–54,473 sequences assigned to Chrysophyceae, comprising 2%–92% of sequences in each sample. Trophic functions were assigned to 76% of ASVs representing 95% of sequences. Of these, 971 ASVs encompassing 4,099,386 sequences (65% of the dataset) were assigned to algae (Figure 2), whose sunlight‐dependent nutrition distinguishes them as preserved groups unlikely to grow in the sediment column in situ. Dinoflagellates and other mixotrophs were abundant in top sediment layers. Apicomplexa (13% of sequences) followed by Fungi (10%) were the second and third most abundant subdivisions, particularly in the sediments of fertilised sites. FIGURE S3. RV coefficients measuring the multivariate correlation between monitoring and paleogenetic algal community times series at the level of genera for L226N (n = 10), L226S (n = 6), L227 (n = 12), L224 (n = 9), and L373 (n = 5). Asterisks indicate the level of statistical significance: p < 0.05 (*), p ≤ 0.01 (**). FIGURE S4. RV coefficients measuring the multivariate correlation between monitoring and paleogenetic records for prevalent algal subdivisions for L226N (n = 10), L226S (n = 6), L227 (n = 12), L224 (n = 9), and L373 (n = 5). Assemblages were compared at the highest levels of t [file EMI-27-e70159-s003.pdf]

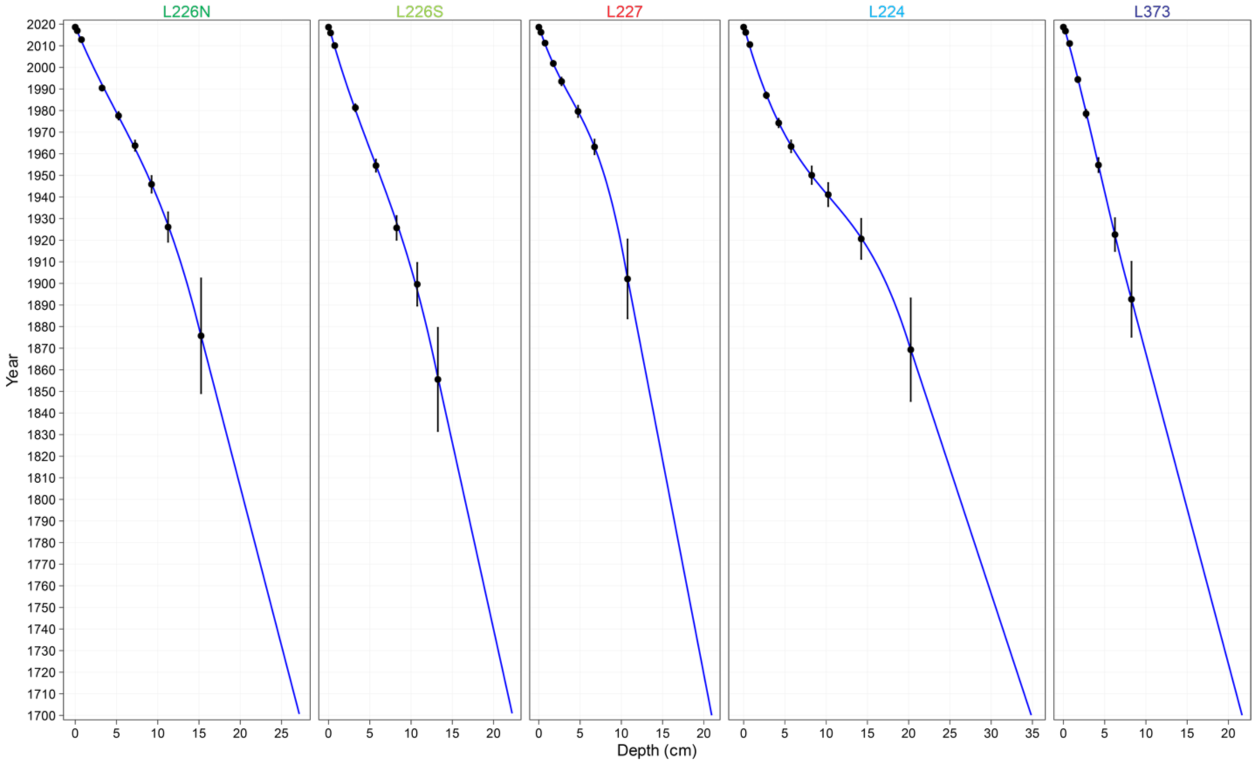

Figure S1. Sediment dating estimated using the constant rate of supply model. Sediment interval midpoint depths are along the x-axis. Vertical bars indicate model error margins. Blue curves show model-interpolated sediment age-dates.

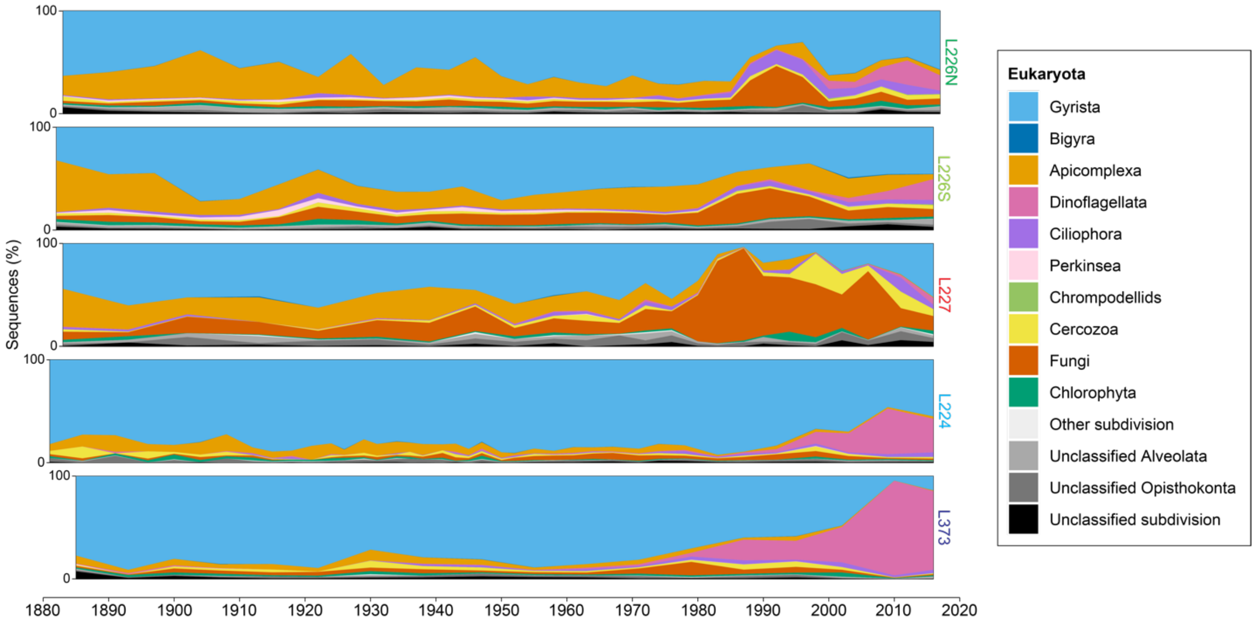

Figure S2. Taxonomic composition of microeukaryotic assemblages in 0.5-cm sediment core intervals of experimental and unmanipulated lakes, extending to the *circa* 1880 preindustrial background for the region. In total, 3,953 18S rRNA gene amplicon sequence variants (ASVs) encompassing 6,345,459 sequences were inferred across 135 sediment samples spanning the five cores. Gyrista was by far the most abundant eukaryotic subdivision in terms of total sequences (61%) and ASVs (754) across samples. Each sediment layer contained 1,645–54,473 sequences assigned to Chrysophyceae, comprising 2–92% of sequences in each sample. Trophic functions were assigned to 76% of ASVs representing 95% of sequences. Of these, 971 ASVs encompassing 4,099,386 sequences (65% of the dataset) were assigned to algae (**Figure 2**), whose sunlight-dependent nutrition distinguishes them as preserved groups unlikely to grow in the sediment column *in situ*. Dinoflagellates and other mixotrophs were abundant in top sediment layers. Apicomplexa (13% of sequences) followed by Fungi (10%) were the second and third most abundant subdivisions, particularly in the sediments of fertilized sites.

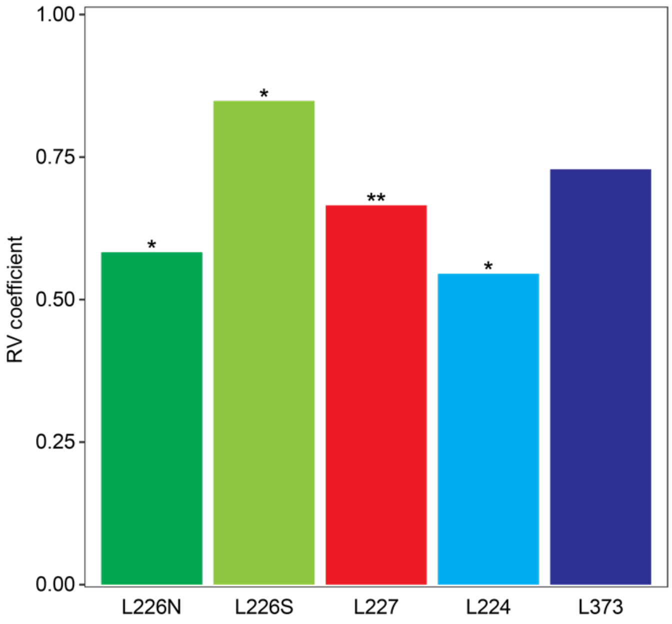

Figure S3. RV coefficients measuring the multivariate correlation between monitoring and paleogenetic algal community times series at the level of genera for L226N (n = 10), L226S (n = 6), L227 (n = 12), L224 (n = 9), and L373 (n = 5). Asterisks indicate the level of statistical significance: $p < 0.05$ (*), $p \leq 0.01$ (**).

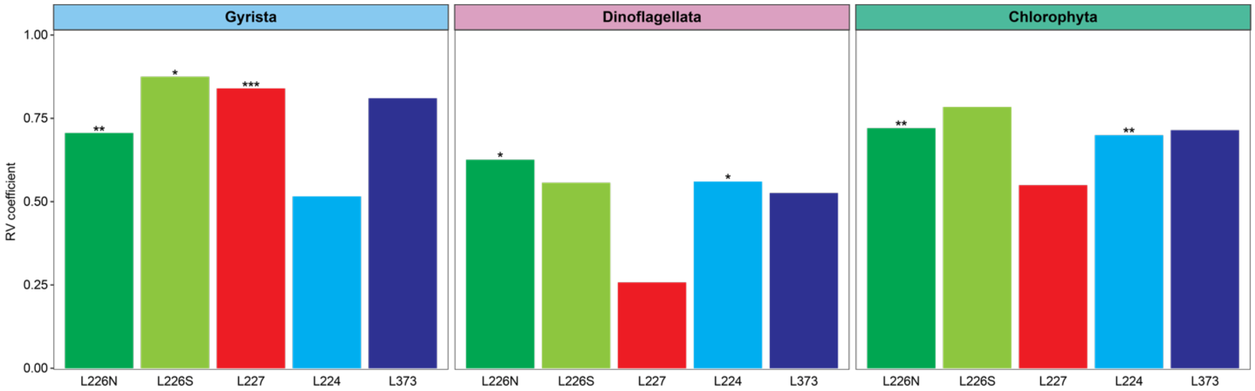

Figure S4. RV coefficients measuring the multivariate correlation between monitoring and paleogenetic records for prevalent algal subdivisions for L226N (n = 10), L226S (n = 6), L227 (n = 12), L224 (n = 9), and L373 (n = 5). Assemblages were compared at the highest levels of taxonomic resolution. Asterisks indicate the level of statistical significance: $p < 0.05$ (*), $p \leq 0.01$ (**), $p \leq 0.001$ (***).

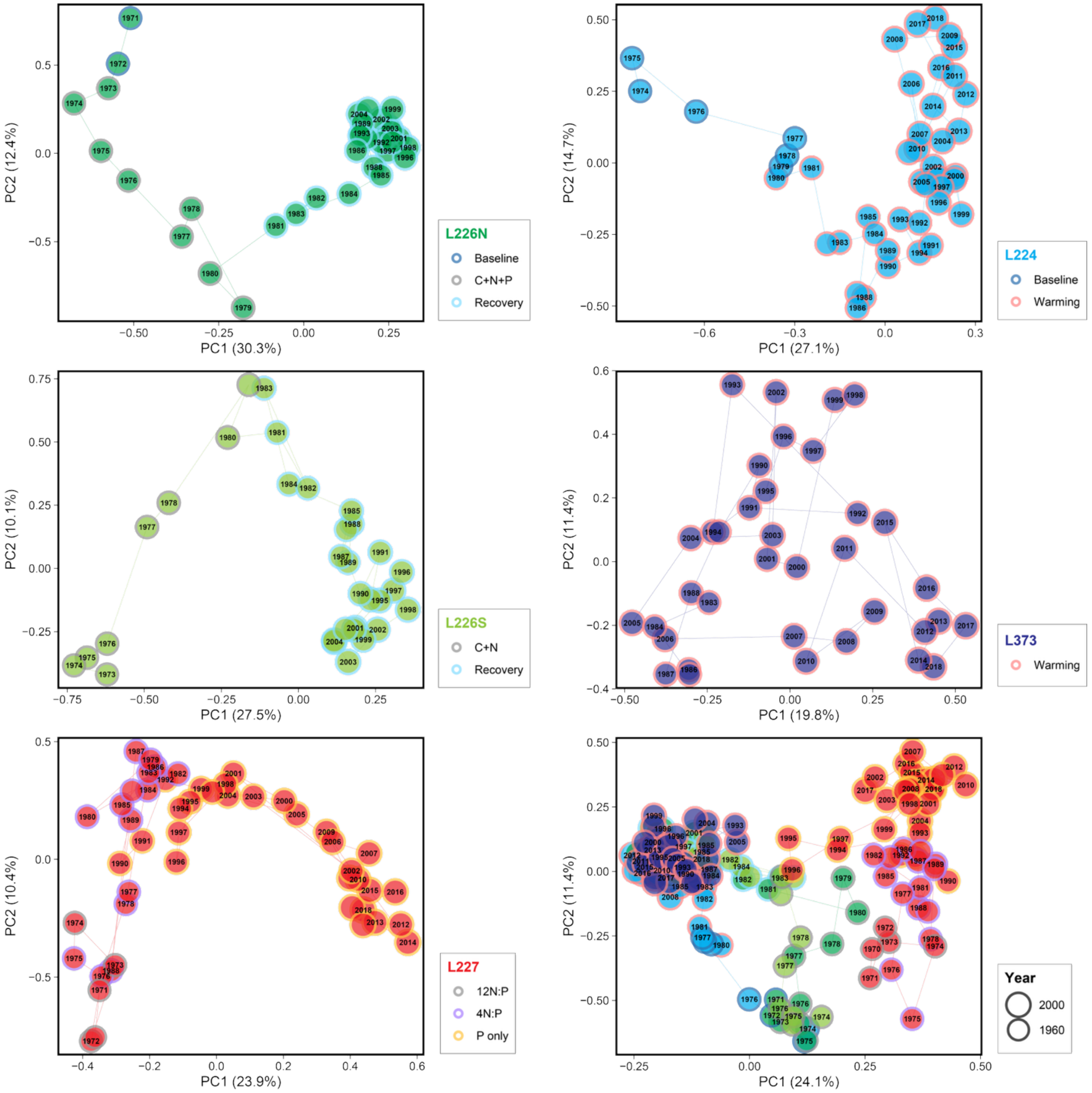

Figure S5. Principal component analyses conducted on monitoring assemblages in individual (all except bottom-right plot) and combined (bottom-right plot) sites. Point labels and sizes indicate the monitoring year. Point colours indicate the site. Contour colours indicate specific nutrient loading regimes, recovery, and warming in each site.

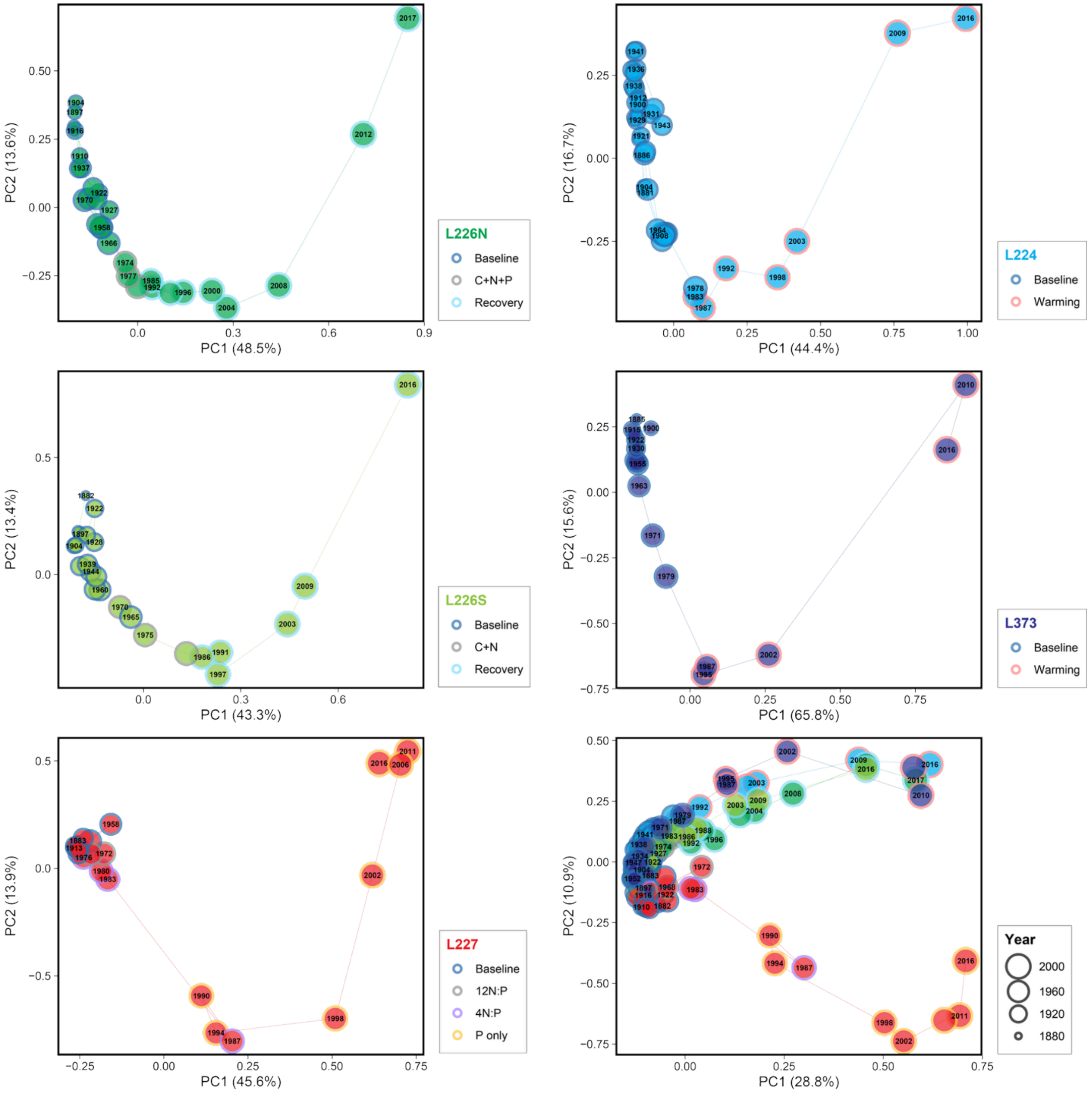

Figure S6. Principal component analyses conducted on paleogenetic assemblages in individual (all except bottom-right plot) and combined (bottom-right plot) sites. Point labels and sizes indicate the sediment interval midpoint estimated year. Point colours indicate the site. Contour colours indicate baseline/pre-manipulation conditions, specific nutrient loading regimes, recovery, and warming in each site.

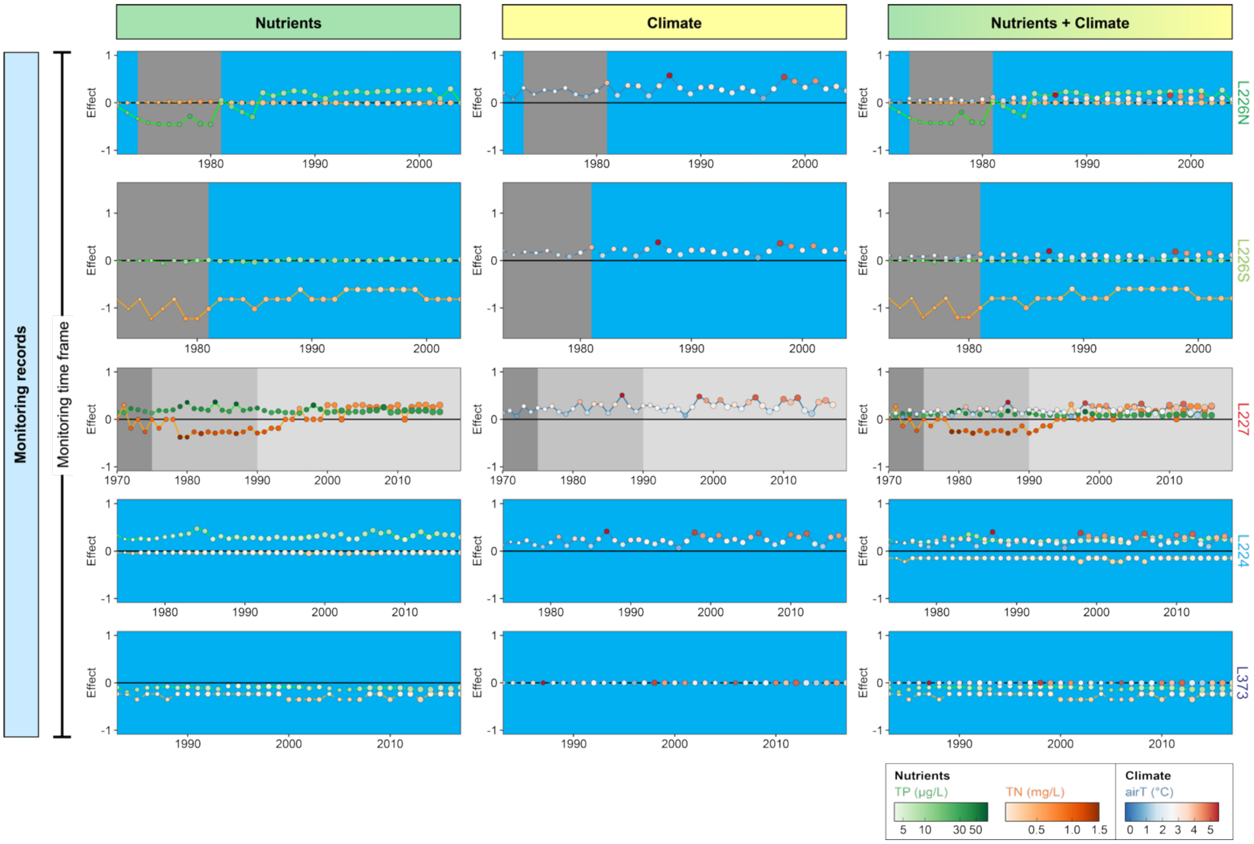

Figure S7. Partial effects over time of individual predictors (TP in green; TN in orange; air temperature in blue to red gradient) on the taxonomic variation in monitoring assemblages fitted in GAMs. Grey panels show experimental regimes in L226N, L226S. and L227 (blue panels indicate eras with no manipulation).

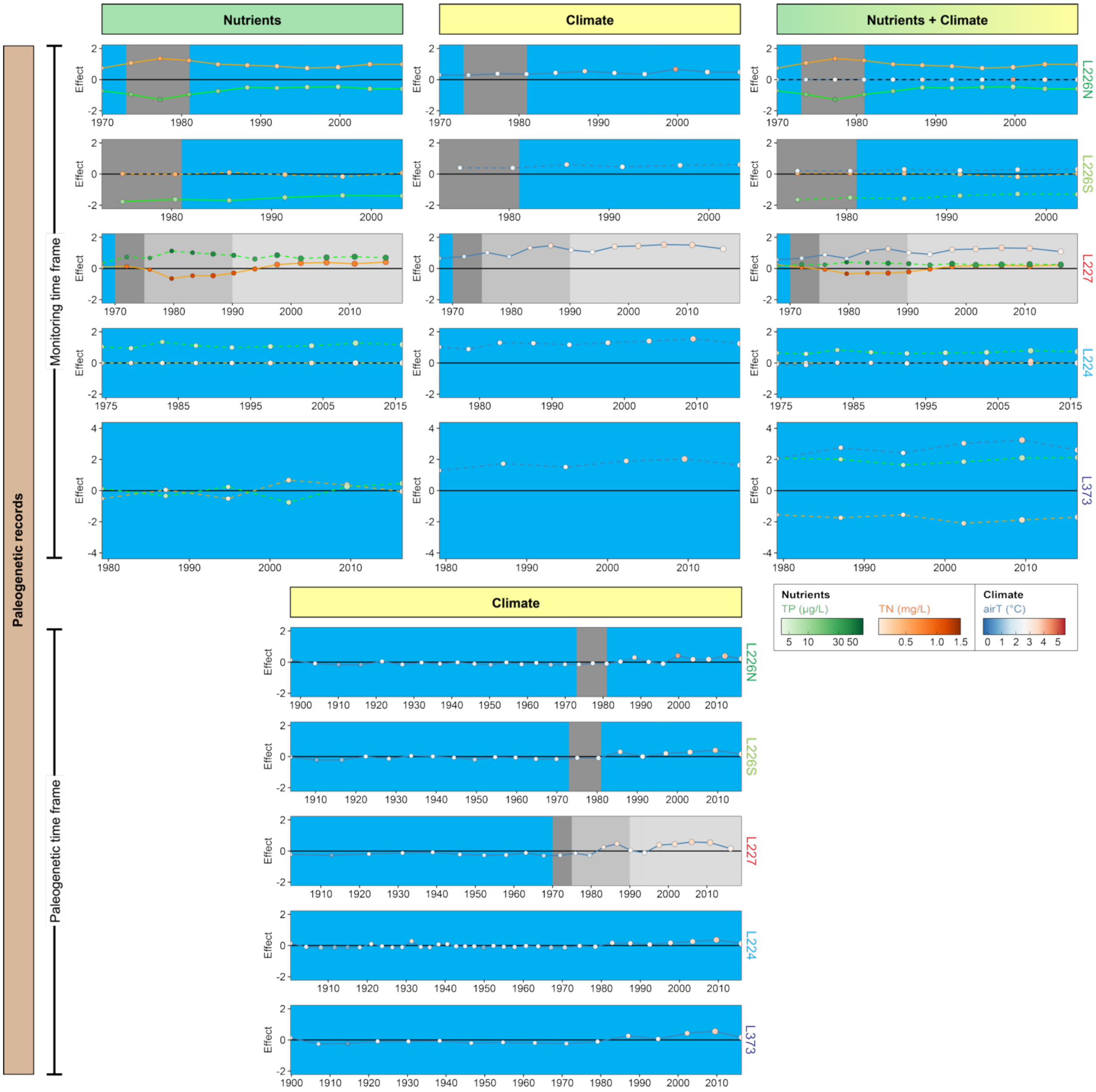

Figure S8. Partial effects over time of individual predictors (TP in green; TN in orange; air temperature in blue to red gradient) on the taxonomic variation in paleogenetic assemblages fitted in GAMs. Separate contribution plots are displayed for GAMs based on paleogenetic records within monitoring or paleogenetic time frames. Grey panels show experimental regimes in L226N, L226S, and L227 (blue panels indicate eras with no manipulation).

Table S2. Monitoring time series harmonization within corresponding sediment intervals.

Site	Sediment interval lower (cm)	Sediment interval upper (cm)	Sediment interval lower year	Sediment interval upper year	Monitoring start year	Monitoring end year
L226N	2.0	1.5	2001.75	2005.83	2002	2004
L226N	2.5	2.0	1997.8	2001.75	1998	2001
L226N	3.0	2.5	1993.94	1997.8	1994	1997
L226N	3.5	3.0	1990.17	1993.94	1990	1993
L226N	4.0	3.5	1986.45	1990.17	1986	1989
L226N	4.5	4.0	1982.77	1986.45	1983	1985
L226N	5.0	4.5	1979.11	1982.77	1979	1982
L226N	5.5	5.0	1975.45	1979.11	1975	1978
L226N	6.0	5.5	1971.77	1975.45	1972	1974
L226N	6.5	6.0	1968.05	1971.77	1971	1971
L226S	1.5	1.0	2000.09	2006.24	2000	2004
L226S	2.0	1.5	1994.2	2000.09	1994	1999
L226S	2.5	2.0	1988.53	1994.2	1989	1993
L226S	3.0	2.5	1983.04	1988.53	1983	1988
L226S	3.5	3.0	1977.71	1983.04	1978	1982
L226S	4.0	3.5	1972.49	1977.71	1973	1977
L227	0.5	0.0	2013.41	2018	2013	2018
L227	1.0	0.5	2008.38	2013.41	2008	2012
L227	1.5	1.0	2003.8	2008.38	2004	2007
L227	2.0	1.5	1999.6	2003.8	2000	2003
L227	2.5	2.0	1995.7	1999.6	1996	1999
L227	3.0	2.5	1992.01	1995.7	1992	1995
L227	3.5	3.0	1988.46	1992.01	1988	1991
L227	4.0	3.5	1984.97	1988.46	1985	1987
L227	4.5	4.0	1981.44	1984.97	1981	1984
L227	5.0	4.5	1977.81	1981.44	1978	1980
L227	5.5	5.0	1973.99	1977.81	1974	1977
L227	6.0	5.5	1969.9	1973.99	1970	1973
L224	0.5	0.0	2012.68	2018	2013	2018
L224	1.0	0.5	2006.4	2012.68	2006	2012
L224	1.5	1.0	2000.51	2006.4	2001	2005
L224	2.0	1.5	1995.02	2000.51	1995	2000
L224	2.5	2.0	1989.88	1995.02	1990	1994
L224	3.0	2.5	1985.08	1989.88	1985	1989
L224	3.5	3.0	1980.6	1985.08	1981	1984
L224	4.0	3.5	1976.42	1980.6	1976	1980
L224	4.5	4.0	1972.51	1976.42	1974	1975
L373	0.5	0.0	2013.03	2018	2013	2018
L373	1.0	0.5	2005.97	2013.03	2006	2012
L373	1.5	1.0	1998.6	2005.97	1999	2005
L373	2.0	1.5	1990.98	1998.6	1991	1998
L373	2.5	2.0	1983.14	1990.98	1983	1990

Table S3. Summary of Pearson correlations between the primary axis of variation (PC1) and time for monitoring or paleogenetic records in each site. "n" is the number of data points.

Records	Site	Pearson r	p-value	n
monitoring	L226N	0.87	2.39E-11	34
monitoring	L226S	0.83	5.25E-09	32
monitoring	L227	0.93	1.28E-22	49
monitoring	L224	0.80	5.64E-11	45
monitoring	L373	0.74	1.88E-07	36
paleogenetics	L226N	0.77	6.16E-07	30
paleogenetics	L226S	0.83	3.91E-07	24
paleogenetics	L227	0.75	2.28E-05	24
paleogenetics	L224	0.73	1.75E-07	39
paleogenetics	L373	0.72	8.42E-04	18

Table S4. Summary of ANOSIM results evaluating the global differences in monitoring or paleogenetic assemblages between sequential periods (selected from baseline/pre-manipulation, specific nutrient loading regime, warming, and recovery) in each site.

Records	Site	Groups	R	p-value	Significance level
monitoring	L226N	Baseline|C+N+P|Recovery	0.885	0.001	***
monitoring	L226S	C+N|Recovery	0.786	0.001	***
monitoring	L227	12N:P|4N:P|P only	0.503	0.001	***
monitoring	L224	Baseline|Warming	0.566	0.003	**
paleogenetics	L226N	Baseline|C+N+P|Recovery	0.572	0.001	***
paleogenetics	L226S	Baseline|C+N|Recovery	0.657	0.001	***
paleogenetics	L227	Baseline|12N:P|4N:P|P only	0.509	0.001	***
paleogenetics	L224	Baseline|Warming	0.765	0.001	***
paleogenetics	L373	Baseline|Warming	0.894	0.001	***

Table S6. Summary of generalized additive model parameters and results. Nonlinear terms are indicated by s(). "n" is the number of data points.

Driver	Records	Time frame	Site	Terms	n	Variable p-values	Deviance explained	AIC
nutrients	monitoring	monitoring	L226N	s(TP), s(TN)	34	TP|5e-11, TN|0.263	0.734	-17.4
nutrients	monitoring	monitoring	L226S	s(TP), TN	31	TN|0.001, TP|0.246	0.353	11.3
nutrients	monitoring	monitoring	L227	TP, s(TN)	47	TP|0.239, TN|5e-7	0.544	1.8
nutrients	monitoring	monitoring	L224	TP, TN	44	TP|0.177, TN|0.900	0.044	15.1
nutrients	monitoring	monitoring	L373	TP, TN	35	TP|0.593, TN|0.257	0.056	13.7
nutrients	paleogenetic	monitoring	L226N	TP, TN	11	TP|0.007, TN|0.0268	0.622	-11.4
nutrients	paleogenetic	monitoring	L226S	TP, s(TN)	6	TP|0.025, TN|0.058	0.997	-31
nutrients	paleogenetic	monitoring	L227	TP, s(TN)	13	TP|0.157, TN|0.009	0.662	8
nutrients	paleogenetic	monitoring	L224	TP, s(TN)	9	TP|0.320, TN|0.779	0.141	10.1
nutrients	paleogenetic	monitoring	L373	s(TP), s(TN)	6	TP|0.066, TN|0.054	0.985	-7.6
climate	monitoring	monitoring	L226N	airT	34	airT|0.034	0.132	19.3
climate	monitoring	monitoring	L226S	airT	32	airT|0.200	0.054	21.7
climate	monitoring	monitoring	L227	airT	48	airT|0.017	0.117	25.2
climate	monitoring	monitoring	L224	airT	44	airT|0.035	0.102	10.3
climate	monitoring	monitoring	L373	s(airT)	35	airT|1	0	11.7
climate	paleogenetic	monitoring	L226N	airT	11	airT|0.027	0.435	-8.9
climate	paleogenetic	monitoring	L226S	airT	6	airT|0.130	0.474	-5.2
climate	paleogenetic	monitoring	L227	airT	13	airT|0.001	0.63	4.2
climate	paleogenetic	monitoring	L224	airT	9	airT|0.115	0.317	8.1
climate	paleogenetic	monitoring	L373	airT	6	airT|0.194	0.378	9.1
climate	paleogenetic	paleogenetic	L226N	s(airT)	28	airT|0.0002	0.508	-4.9
climate	paleogenetic	paleogenetic	L226S	s(airT)	21	airT|0.001	0.502	-0.7
climate	paleogenetic	paleogenetic	L227	s(airT)	22	airT|5e-7	0.741	-2.1
climate	paleogenetic	paleogenetic	L224	s(airT)	35	airT|0.0006	0.359	-4.3
climate	paleogenetic	paleogenetic	L373	s(airT)	16	airT|0.002	0.593	6.3
nutrients + climate	monitoring	monitoring	L226N	airT, s(TP), s(TN)	34	airT|0.297, TP|4e-10, TN|0.991	0.737	-16.8
nutrients + climate	monitoring	monitoring	L226S	TN, airT, s(TP)	31	TN|0.002, airT|0.447, TP|0.284	0.36	12.5
nutrients + climate	monitoring	monitoring	L227	TP, airT, s(TN)	47	TP|0.527, airT|0.035, TN|1e-5	0.531	2.2
nutrients + climate	monitoring	monitoring	L224	TP, TN, airT	44	TP|0.302, TN|0.565, airT|0.049	0.133	12.8
nutrients + climate	monitoring	monitoring	L373	TP, TN, s(airT)	35	TP|0.593, TN|0.257, airT|1	0.056	13.7
nutrients + climate	paleogenetic	monitoring	L226N	TP, TN, s(airT)	11	TP|0.007, TN|0.027, airT|0.860	0.622	-11.4
nutrients + climate	paleogenetic	monitoring	L226S	TP, s(TN), airT	6	TP|0.999, airT|0.999, TN|1	1	-159.1
nutrients + climate	paleogenetic	monitoring	L227	TP, s(TN), airT	13	TP|0.411, airT|0.002, TN|0.034	0.868	-2.9
nutrients + climate	paleogenetic	monitoring	L224	TP, s(TN), s(airT)	9	TP|0.571, TN|0.846, airT|0.255	0.244	10.2
nutrients + climate	paleogenetic	monitoring	L373	TP, TN, airT	6	TP|0.421, TN|0.601, airT|0.340	0.638	9.9
